# The Origin of GPCRs: Identification of Mammalian like *Rhodopsin*, *Adhesion*, *Glutamate* and *Frizzled* GPCRs in Fungi

**DOI:** 10.1371/journal.pone.0029817

**Published:** 2012-01-04

**Authors:** Arunkumar Krishnan, Markus Sällman Almén, Robert Fredriksson, Helgi B. Schiöth

**Affiliations:** Department of Neuroscience, Functional Pharmacology, Uppsala University, Uppsala, Sweden; University of Medicine & Dentistry of New Jersey - New Jersey Medical School, United States of America

## Abstract

G protein-coupled receptors (GPCRs) in humans are classified into the five main families named *Glutamate*, *Rhodopsin*, *Adhesion*, *Frizzled* and *Secretin* according to the GRAFS classification. Previous results show that these mammalian GRAFS families are well represented in the Metazoan lineages, but they have not been shown to be present in Fungi. Here, we systematically mined 79 fungal genomes and provide the first evidence that four of the five main mammalian families of GPCRs, namely *Rhodopsin*, *Adhesion*, *Glutamate* and *Frizzled*, are present in Fungi and found 142 novel sequences between them. Significantly, we provide strong evidence that the *Rhodopsin* family emerged from the cAMP receptor family in an event close to the split of Opisthokonts and not in Placozoa, as earlier assumed. The *Rhodopsin* family then expanded greatly in Metazoans while the cAMP receptor family is found in 3 invertebrate species and lost in the vertebrates. We estimate that the *Adhesion* and *Frizzled* families evolved before the split of Unikonts from a common ancestor of all major eukaryotic lineages. Also, the study highlights that the fungal *Adhesion* receptors do not have N-terminal domains whereas the fungal *Glutamate* receptors have a broad repertoire of mammalian-like N-terminal domains. Further, mining of the close unicellular relatives of the Metazoan lineage, *Salpingoeca rosetta* and *Capsaspora owczarzaki*, obtained a rich group of both the *Adhesion* and *Glutamate* families, which in particular provided insight to the early emergence of the N-terminal domains of the *Adhesion* family. We identified 619 Fungi specific GPCRs across 79 genomes and revealed that Blastocladiomycota and Chytridiomycota phylum have Metazoan-like GPCRs rather than the GPCRs specific for Fungi. Overall, this study provides the first evidence of the presence of four of the five main GRAFS families in Fungi and clarifies the early evolutionary history of the GPCR superfamily.

## Introduction

G protein-coupled receptors (GPCRs) are the largest family of transmembrane receptors with about 800 members in the human genome serving as targets for many drugs [1], [2]. Over the years, the repertoire of GPCRs has been analyzed in several species which are from the phyla Chordata, Echinodermata, Arthropoda, Nematoda, Cnidaria, Placozoa and Amoebozoa [3]–[8]. At first, in 1994, Attwood and Findlay categorized the superfamily into six classes (A–F) based on sequence homology and functional similarity [9]. Later, our comprehensive phylogenetic analysis of the human repertoire provided the GRAFS classification. This system grouped the mammalian GPCR repertoire into five main families; *Glutamate* (Class C), *Rhodopsin* (Class A), *Adhesion* (Class B), *Frizzled* (Class F), and *Secretin* (Class B) [10]. The *Rhodopsin* family is the largest with 683 members in humans [8], characterized by short N-termini and interactions with a broad variety of ligands. The *Glutamate* family is distinguished with long N-termini which act as the endogenous ligand binding region. The *Adhesion* receptors have long N-termini which contain a plethora of multiple domains while the *Frizzled* receptors have long cysteine-rich N-termini [1].

Mining of GPCRs has revealed that each of the five main mammalian families have long evolutionary histories. Recently, our group has shown the connections between the GPCR families and provided strong evidence that the *Rhodopsin*, *Adhesion*, *Frizzled* and *Secretin* share a common origin with the cAMP receptors (Class E) and dismissed relationship with families like the insect odorant receptors, insect gustatory receptors, trehalose receptors and DUF267, which in many publications were considered to be GPCRs [8]. Our previous study [8] also showed an early presence of *Rhodopsin* (7tm_1) family members in the phylum Placozoa (*Trichoplax adhaerens*), an invertebrate which forms the basal group of Metazoa [11], while *Adhesion* (7tm_2) and *Frizzled* family members were found in the phylum Amoebozoa (*Dictyostelium discoideum*), which is a basal group to both Fungi and Metazoa [12]. The *Glutamate* (7tm_3) family members were found in the phylum Heterokontophyta (*Thalassiosira pseudonana*), a diatom classified under the Chromalveolata kingdom [13] while the earliest occurrence of *Secretin* family members, which evolved from *Adhesion* receptor family [14], was found in the phylum Nematoda (*Caenorhabditis elegans*) [15]. Further, the cAMP receptor family, predicted to be the ancestor to the main families (Class A, B and F) is present only in invertebrates within the Metazoan lineage [8], [16].

Recently, a multi-taxon genome sequencing initiative endorsed by National Human Genome Research Institute (NHGRI) shed light on how multicellularity evolved. The study suggests that the two main eukaryotic kingdoms- Metazoa and Fungi along with unicellular relatives like Choanoflagellata and Filasterea share a common origin or group together under a eukaryotic supergroup known as Opisthokonta [17]. Over the years, GPCR repertoires have been particularly well studied in Metazoa as compared to Fungi. The five main GPCR families have previously not been found in Fungi, despite the presence of *Adhesion*, *Frizzled* and *Glutamate* family members in *D. discoideum* [8]. Literature reports that GPCR homologues already identified in Fungi can be classified into at least six classes or families that do not belong to the mammalian GRAFS families. The six classes include pheromone receptors sensing peptide pheromones (Ste2), pheromone receptors sensing lipid modified peptide pheromones (Ste3), nutrient sensors (Gpr1), Stm1-like nitrogen sensors (Stm1), microbial opsins (Nop-1 and Orp-1) and cAMP like receptors [18]. Until today, a few GPCR homologues have been identified in *Saccharomyces cerevisiae*, *Aspergillus nidulans*, *Cryptococcus neoformans*, *Magnaporthe grisea* and in *Neurospora crassa*, but they are categorized under the known six classes of fungal GPCRs and not among the GRAFS families [18]–[21].

GPCRs in Fungi were proposed to have a role in fungal-plant interactions. They are involved in recognizing various signal molecules or ligands from plant cells paving the way for the Fungi to destroy valuable crops [22]. Pathogenic Fungi are also responsible for causing diseases in humans like Histoplasmosis and severe Pneumonia in people with immune system problems [23], [24]. Recent advances in sequencing technologies led to a remarkable increase in the number of fully sequenced fungal genomes. The Fungal genome initiative (FGI) endorsed by Broad institute at MIT (http://www.broad.mit.edu/annotation/fgi) and DOE Joint Genome Institute (JGI; http://www.jgi.doe.gov/), sequenced several fungal genomes that are pathogenic to humans and plants.

In this study, we have mined for GPCRs in a complete proteome dataset of Fungi from UniProt and over 18 genomes of Fungi that are available at FGI (Broad institute). We have also investigated genomes belonging to eukaryotic lineages like Choanoflagellata (*Salpingocea rosetta*), Filasterea (*Capsaspora owczarzaki*), which share a common origin with Metazoa and from Alveolata (*Paramecium tetraurelia* and *Tetrahymena thermophila*). Overall, we systematically mined for GPCRs in 83 genomes to obtain a nearly complete set of GPCRs in Fungi and unicellular Metazoan relatives.

## Results

### Identification of novel homologues of the *Rhodopsin*, *Adhesion*, *Glutamate* and *Frizzled* like receptors in Fungi and major eukaryotic lineages

We searched for GPCRs in a comprehensive protein sequence dataset that comprise proteomes of several species representing the eukaryotic lineages like Fungi (79 species), Choanoflagellata (*S. rosetta*), Filasterea (*C. owczarzaki*), Alveolata (*P. tetraurelia*, *T. thermophila*) and from collection of proteins (3465 proteins) from several species that are from the phylum Porifera, which is one of the most basal Metazoan lineages. The complete dataset was searched using Hidden Markov Models (HMM) models for the 11912 families of the Pfam database (version 24), which revealed that the characteristic Metazoan GPCR families of the GRAFS classification are wide spread in the eukaryotic domain. This study identified 142 novel sequences distributed among the *Rhodopsin*, *Adhesion*, *Glutamate* and *Frizzled* families that have not previously been reported in the Fungi kingdom. In *S. rosetta*, 8 novel members of the *Adhesion* and 1 member of the *Glutamate* family were found. In *C. owczarzaki*, 7 novel sequences of the *Adhesion* and 20 for the *Glutamate* family were found.

Also, one sole representative was found for the *Glutamate* family in Porifera. In addition to the GRAFS families, our study identified 57 cAMP receptor family sequences in Fungi, 8 in Alveolata, and more surprisingly 1 cAMP receptor each in *Branchiostoma floridae* and *Lottia gigantea*. All identified novel sequences in individual species are listed with their accession numbers (Table S1); FASTA sequences are provided in Dataset S1 and the list of species investigated is given in Table S2.

### The *Rhodopsin* and cAMP receptor family

The *Rhodopsin* family of GPCRs constitutes the largest family of GPCRs in vertebrates with 683 members in humans, classified into four main groups, termed α-, β-, γ-, and δ-group, and 13 major subfamilies [10]. The *Rhodopsin* family is very well represented in both vertebrates and in invertebrates with the ancient members (343 receptors) was found in *T. adhaerens*. On the other hand, the cAMP receptor family in *D. discoideum* is found in plants and in Alveolata [8]. Also, they are present in Fungi kingdom, but found in very few invertebrate species and subsequently lost in the vertebrates within the Metazoan lineages [8], [17].

#### Novel homologues

This study identified 12 novel sequences of the *Rhodopsin* family in Fungi. However, the sequences were only found in 3 species: *Allomyces macrogynus* (3 sequences) from Phylum Blastocladiomycota, *Batrachochytrium dendrobatidis* (1 sequence) and *Spizellomyces punctatus* (8 sequences) which belong to the phylum Chytridiomycota, known to be a basal fungal lineage and found to have split prior to the Dikarya subkingdom. Similar to the previous reports, our search did not identify *Rhodopsin* family members in Dikarya that includes the two major phyla Ascomycota and Basidiomycota of Fungi. Further, search for the *Rhodopsin* family members in Choanoflagellata, Filasterea, Alveolata and Porifera failed to find homologues.

#### Conserved features

**Figure 1:**
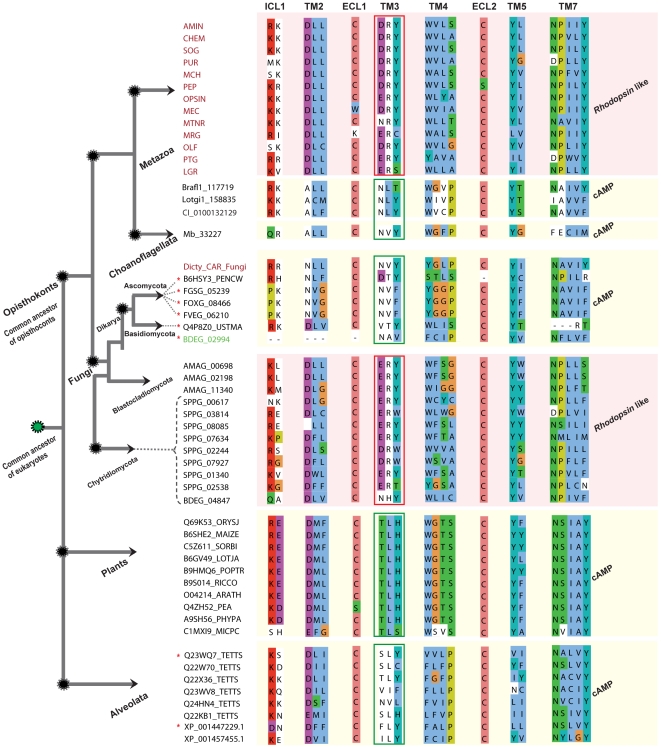
Alignment showing shared and group specific motifs between Rhodopsin and cAMP like receptors in eukaryotes. We include consensus sequences obtained for each of the known 13 subgroups (colored red) of the large *Rhodopsin* family members from *H. sapiens* and a lone cAMP member from *C. intestinalis* to represent the Metazoan lineage. The alignment includes the identified cAMP like sequences from *B. floridae* (Brafl1_117719), *L. gigantea* (Lotgi1_158835), *M. brevicollis* (Mb_33227) a Choanoflagellata, consensus sequence obtained from 51 cAMP receptors from Fungi (Dicty_CAR_Fungi), and Alveolata (8) and *Rhodopsin* like sequences found only from 3 species, *A. macrogynus* (3) from Blastocladiomycota, *B. dendrobatidis* (BDEG_02994) and *S. punctatus* (8) which are member of the Chytridiomycota, an ancestral fungal lineage. The alignment also includes 10 cAMP receptor sequences from plants, obtained from Pfam database. The sequences are grouped based on family as cAMP and *Rhodopsin* like with respective to the major lineages across the eukaryotic tree to display the distinctive and shared motifs between them. The sequences which are marked with asterisk (red) received 7tm_1 (*Rhodopsin* like) as the best domain hit for HMM search against Pfam A families. We grouped those 8 sequences as cAMP receptors based on motifs and with strong support from phylogeny. The sequence (BDEG_02994, colored green) has only 5 TM domains, but was included as a lone representative for cAMP in Chytridiomycota. Major group specific motifs are indicated in red (*Rhodopsin*) and green (cAMP) rectangular boxes respectively. The text above the alignment denotes the transmembrane (TM) passage, intracellular loops (ICL) or extra-cellular loops (ECL). Schematic representation on the left indicates the evolution of major eukaryotic lineages from a unicellular common ancestor. Nodes defining relationships across the eukaryotic tree are marked with dotted circles (black), common eukaryotic ancestor (green), the same representation applies to Figure 4 and 5. The dotted lines pointing to the sequence id indicate to which phylum it belongs to, within the Fungi kingdom. The overall schematic representation of the eukaryotic tree was adapted from [17].

The 12 novel Fungi members of the *Rhodopsin* family share many motifs characteristic for the *Rhodopsin* family in Metazoa, such as aspartate (D) in TM2, D/ERY in TM3, NP in TM7 and cysteine (C) in ELC1 and 2 (Figure 1). In addition, there are motifs that provide a link between the cAMP receptor family and the *Rhodopsin* family like: arginine (R)/lysine (K) in ICL1, aspartate (D) in TM2, tyrosine (Y) in TM3 & 5, cysteine (C) in ELC1 & 2. Further, we found 8 sequences which were initially identified as putative members of the *Rhodopsin* family in the Pfam search. They are distributed as 2 sequences in the phylum Alveolata, 4 in Ascomycota and 1 each in Basidiomycota and Chytridiomycota. These 8 sequences lack the D/ERY motif that is common for the *Rhodopsin* family but instead has motif NxY that are present among the cAMP receptor sequences (Figure 1).

#### Phylogenetic analysis

**Figure 2:**
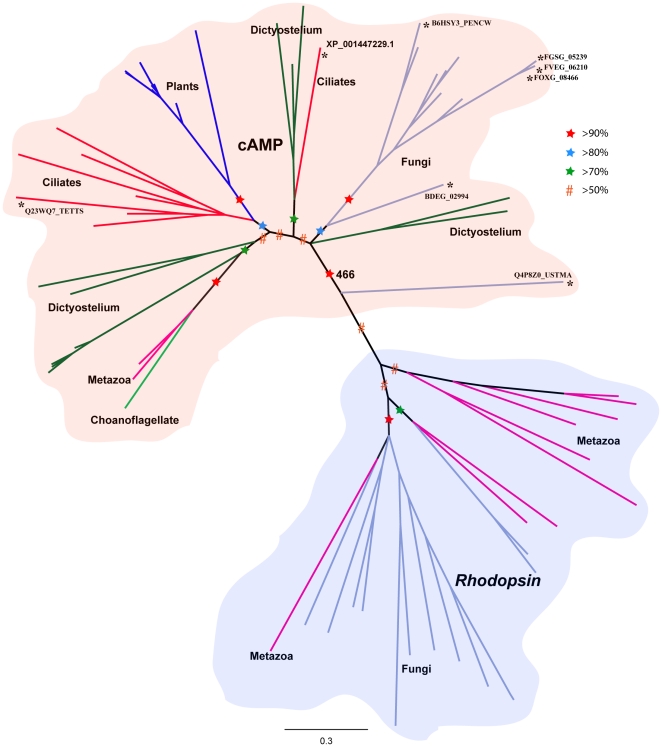
Phylogenetic relationships between the cAMP and Rhodopsin families across eukaryotic lineages. The tree is based on Bayesian method of phylogenetic inference. The phylogenetic tree is based only on the transmembrane region. Robustness of the nodes is tested with posterior probabilities based on MCMC analysis (see Methods). Nodes supported by posterior probabilities between 50–70% are marked with hash symbol (red) and nodes between 70–80%, 80–90% and >90% are marked with a star colored green, blue and red, respectively. Bootstrap support from maximum likelihood approach (PhyML) is indicated for the node that separates *Rhodopsin* and cAMP receptors. The edges marked with asterisk were found to be *Rhodopsin* like sequences (8) in HMM search, but classified as cAMP based on phylogeny and motifs. The motifs of those 8 sequences similar to the other cAMP sequences are shown in Figure 1.

The phylogenetic relationships for the *Rhodopsin* and cAMP receptor family were investigated using the Bayesian approach implemented in MrBayes 3.1.2. Two preliminary trees were constructed: the first tree included the novel *Rhodopsin* family members identified in Fungi (Chytridiomycota and Blastocladiomycota) with the representative sequences from 13 subgroups of the *Rhodopsin* family in humans (Figure S1): the second tree included Fungi (12 sequences) and the representative *Rhodopsin* family members from *T. adhaerens*, which belong to the most basal lineage of Metazoa and therefore containing the evolutionarily most distant members of the *Rhodopsin* family (Figure S2). Both these preliminary trees demonstrated that the Fungi *Rhodopsin* family members cluster together with more than 90% posterior probability (PP) and clearly separate the cluster from its homologues in humans and *T. adhaerens*, respectively. Further, the 8 sequences (identified as *Rhodopsin* family members in Pfam search) that do not have the D/ERY motif and instead have motifs corresponding to the cAMP receptor family, cluster (PP>95%, 367/500) separate from the *Rhodopsin* family members identified in Fungi. Based on this inference, a separate phylogeny was constructed that included all newly identified homologues of the cAMP receptor family and the *Rhodopsin* family with the representative sequences from previous repertoires of both the families (Figure 2). The unrooted tree demonstrated two clusters, one grouping the cAMP receptor family and the other grouping the *Rhodopsin* family.

All newly identified cAMP receptor family sequences in Fungi, Alveolata, Metazoa, Choanoflagellata and the representative sequences in *D. discoideum* and homologues from plants cluster together with a posterior probability greater than 90% (466/500). Furthermore, the tree demonstrated that the ancient cAMP receptor family members identified from Alveolata (ciliates) cluster together (PP>80%) with the cAMP receptors from plants. The 8 sequences identified as *Rhodopsin* family in Pfam search cluster with the cAMP receptor family homologues. Of the 8 sequences, Q23WQ7_TETTS and XP_001447229.1 from ciliates were placed in the branch with the other cAMP receptor family members from ciliates (PP>90%) and *D. discoideum* (PP>70%), respectively (Figure 2). The rest of the sequences were from Fungi (BDEG_02994, FOXG_08466, FVEG_06210, FGSG_05239, B6HSY3_PENCW) and unambiguously clustered with the putative cAMP receptors from Fungi (>80%) with the exception of Q4P8Z0_USTMA, which was placed basal to the node clustering all cAMP receptor family sequences. Instead, we classified the sequence Q4P8Z0_USTMA as a member of the cAMP receptor family based on the characteristic NxY motif in TM3 (Figure 1). All the *Rhodopsin* family members in Fungi cluster (PP>90%) with the sequences in human. However, the sequences in Fungi form a separate cluster (PP>90%) that is distinct from the human sequences, which are representative for 13 Metazoan subgroups of the *Rhodopsin* family.

#### Scatter plot

**Figure 3:**
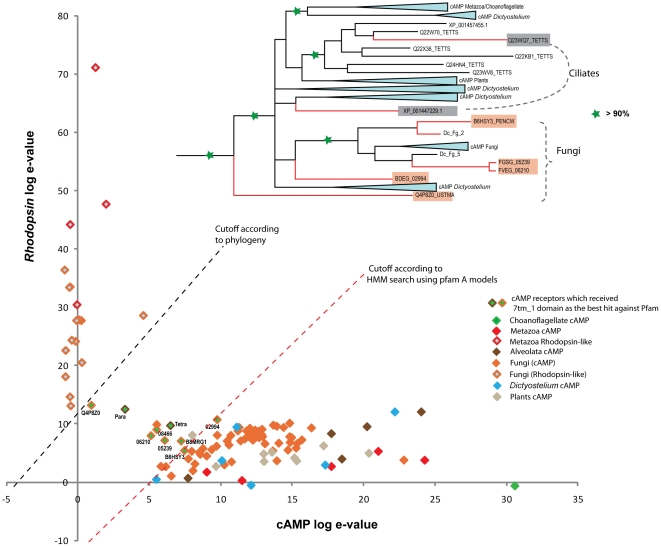
Scatter plot distinguishing cAMP and the Rhodopsin like sequences. The sequences which are plotted were tested with HMM search using 7tm_1 (PF00001) and Dicty_CAR (PF05462) HMM models downloaded from Pfam. The e-values for each *Rhodopsin* like sequence (Y-axis) is plotted against the e-values of cAMP like sequences (X-axis) in a logarithmic scale. The members are distinguished with colors corresponding to each group shown in the right corner. The dotted line in red shows the approximate cutoff which clearly distinguishes cAMP and *Rhodopsin* like sequences according to the HMM search with the HMM models. The special cases which are shown in green with accession id, received e-values very similar in HMM search against both the models, but were classified as *Rhodopsin* like sequences according to the HMM search. Those 8 sequences which belong to ciliates (2) and Fungi (6) were classified as cAMP like sequences with strong support from phylogeny, which is shown as an inset at the top. The posterior probability more than 90% is marked with a star (green). An approximate cutoff according to the phylogeny is shown as dotted lines (black).

The similarity between the *Rhodopsin* (PF00001) and the *Dictyostelium* cyclic AMP receptor: Dicty_CAR (PF05462) was tested using the HMM models downloaded from Pfam database. A scatter plot on a logarithmic scale was made using the e-value for each sequence (used in the phylogeny) aligned with both *Rhodopsin* and Dicty_CAR HMM models using HMMSEARCH (Figure 3). The plot demonstrates that there are uncertain sequences which receive very similar scores but were classified as *Rhodopsin* family based on a negligible difference in e-value which over scored the Dicty_CAR HMM model. However, based on the strong support from phylogeny and sequence motifs, we classified these sequences as cAMP receptor homologues.

### The *Adhesion* receptor family

The *Adhesion* family of GPCRs is the second largest according to the GRAFS GPCR classification with 33 members in humans [3]. The family is characterized with long N-termini containing multiple functional domains and having numerous sites for glycosylation events [1]. Comprehensive phylogenetic analysis on the TM regions of human *Adhesion* receptors classified the family into 8 main groups (I–VIII). The phylogenetic classification was also supported by the composition of the functional domains in the N-termini which differs between the subgroups.

#### Novel homologues

This study identified 32 novel *Adhesion* family members in Fungi. They are distributed as 30 sequences within 22 species of the phylum Ascomycota and 2 sequences in *A. macrogynus* from the phylum Blastocladiomycota. Also, 8 sequences in *S. rosetta*, 7 in *C. owczarzaki* and 1 in *P. tetraurelia* were found. Interestingly, the *Adhesion* family members were not found in the phylum Basidiomycota despite being grouped with the phylum Ascomycota, which has 30 members of the *Adhesion* family.

#### Conserved features

The 48 novel *Adhesion* family members found in major eukaryotic lineages shared motifs of the *Adhesion* family common to known Metazoan sequences. The common motifs/conserved residues were adapted from our previous study on the overall relationship between the GPCR families [8]. Residues cysteine (C) in ECL 1 & 2, tryptophan (W) in TM3, proline (P) in TM4 & 5 and glycine (G) in TM7 were found to be >90% conserved between all eukaryotic lineages in which the *Adhesion* family receptors were identified.

#### Phylogenetic analysis

A phylogenetic tree of the *Adhesion* family was built with the novel members of the family identified from Fungi, Choanoflagellata, Filasterea and Alveolata with the formerly classified sequences from human (Group I–VIII and VLGR1) and representative sequences from *C. intestinalis*, *B. floridae*, *N. vectensis* and *T. adhaerens*, which have key evolutionary position within the Metazoan lineages (Figure S3). The phylogenetic analysis revealed that the *Adhesion* family sequences from Fungi (Ascomycota and Chytridiomycota) clustered separately (PP 99%, 443/500) indicating that were not orthologous to any of the Metazoan receptors, though they have diverged from a common ancestor to all Metazoan and Fungi genes of the *Adhesion* family. Within the largest cluster of Fungi *Adhesion* family members, the sequences from the phylum Ascomycota split into two groups. Furthermore, the sequences from the phylum Blastocladiomycota (AMAG_09540, AMAG_13158 from *A. macrogynus*) clustered with the group of Ascomycota. Interestingly, the lone representative (XP_001450983) of the *Adhesion* family from *T. thermophila* (Alveolata) clustered with the *Adhesion* family members in Fungi with a posterior probability of >95% (272/500). The novel putative members of the *Adhesion* family from *S. rosetta* and *C. owczarzaki* clustered with the representative sequences of the *Adhesion* family from the Metazoan lineage, as they all share a common origin. VLGR1 from human and its orthologue in *N. vectensis* (NV_242264) were placed basal to the node that clustered the Metazoan sequences.

#### N-terminal domain architecture

**Figure 4:**
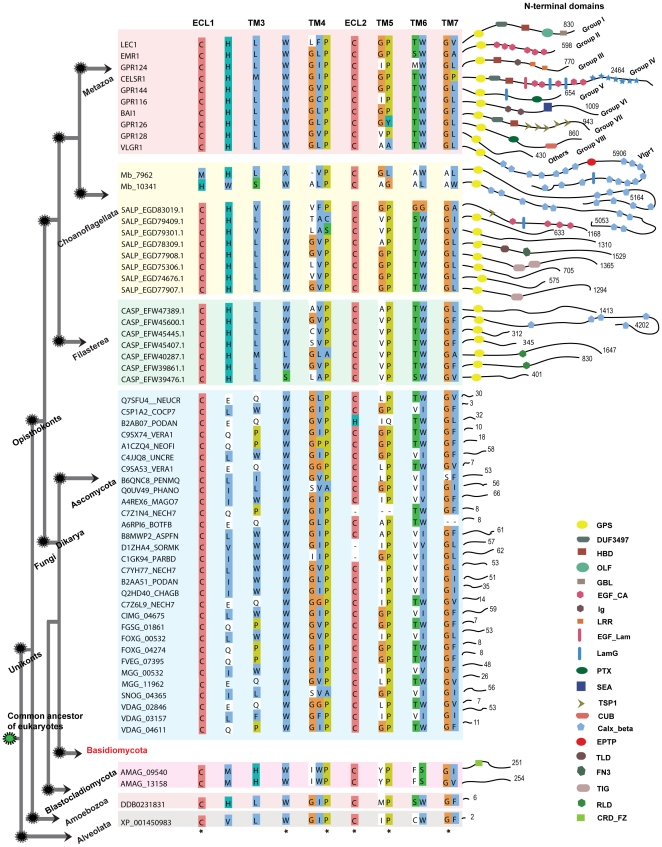
Conserved features and structural motifs within the Adhesion receptor family in eukaryotes. The illustration represents known *Adhesion* receptors from Metazoan lineage with one sequence for each group I–VIII, GPR128 as others and VLGR1. Sequence DDB0231831, only member from *D. discoideum* represents Amoebozoa. The illustration includes newly identified sequences from other eukaryotic lineages like Choanoflagellata (*S. rosetta*; 8), Filasterea (*C. owczarzaki*; 7), Fungi (32), and Alveolata (*P. tetraurelia*; 1). The sequences are grouped respective to the major lineages across the eukaryotic tree. Common motifs of the *Adhesion* receptors were adapted from [8]. The residues which have ≥90% conservation across the eukaryotic tree are marked with a star at the bottom of the alignment. Interestingly, the loss of the *Adhesion* receptors in Basidiomycota phylum which descended from a common ancestor of Dikarya is indicated in red. The right part of the figure displays the diversity within the N-termini of the *Adhesion* receptors. The domains were identified with Pfam search and also verified with RPS-Blast with a cutoff e-value of 0.1. Each domain is marked with a symbol, and the explanation is found in the legend in the lower right corner. The numbers at corner of each connective thread along the domain symbols indicates the length of the N-termini. The following domains were found: GPS (GPCR proteolytic site), DUF3497 (Domain of unknown function), HBD (hormone-binding domain), OLF (olfactomedin domain), GBL (galactose-binding lectin domain), EGF_CA (calcium-binding epidermal growth factor-like domain), Ig (immunoglobulin domain), LRR (leucine-rich repeat), CA (cadherin repeats), EGF_Lam (laminin type epidermal growth factor domain), LamG (laminin G domain), Pentaxin domain, SEA (sea urchin sperm protein domain), TSP1 (thrombospondin repeats, type 1), CUB (C1r/C1s urinary epidermal growth factor and bone morphogenetic domain), Calx-beta domain, EPTP (epitempin protein domain), TLD (this domain is predicted to be an enzyme and is often found associated with pfam0147), FN3 (Fibronectin type III domain), TIG (this family consists of a domain that has an immunoglobulin like fold), RLD (Receptor L domain), CRD_FZ (CRD_domain cysteine-rich domain, also known as Fz (frizzled) domain).

The N-terminal domains of all newly identified *Adhesion* family members were searched and presented together with abbreviations in Figure 4. The N-terminal domain architecture for the representative sequences from group I–VIII in human and for two sequences MB_7962 and MB_10341 from *M. brevicollis* are thoroughly described in our previous report [14].

*Adhesion* family members identified in *S. rosetta* and *C. owczarzaki* have long N-termini ranging from 312–4202 amino acid residues, comparative to the N-termini of the *Adhesion* family receptors in human, which range from 430–5906 residues. The sequences in *S. rosetta* and *C. owczarzaki* have a GPS domain characteristic for the *Adhesion* family members, which is not found in any other GPCR family. One exception is SALP_EGD83019.1 which lacks the GPS domain but has 4 EGF_CA domains, which are present in the sequence of EMR1 (Group II) and CELSR1 (Group III) in human. The same sequence also has one Tsp1 domain, which is found in sequence BAI1 (Group VII) and LamG domain found in sequences CELSR1 (Group III) and GPR144 (Group V). Furthermore, CASP_EFW45600.1 in *C. owczarzaki* has five Calx_beta domains, which are a signature for VLGR1 (the very long G protein-coupled receptors) in Metazoa. This may suggest that CASP_EFW45600.1 is a putative orthologue to VLGR1 in human. Several domains were identified that have not been reported previously in the *Adhesion* family like the TIG domain in the sequences SALP_EGD77907.1, SALP_EGD77908.1; the TLD domain in sequence SALP_EGD78309.1 from *S. rosetta* and the RLD domain in sequences CASP_EFW40287.1 and CASP_EFW39861.1 from *C. owczarzaki*. Interestingly, the 30 putative *Adhesion* family members in phylum Ascomycota of Fungi and 1 member in Alveolata have short N-termini ranging from 2–66 residues with no functional domains. But the *Adhesion* receptors (AMAG_09540 and AMAG_13158) identified in *A. macrogynus*, belonging to the basal fungal lineage Blastocladiomycota, has an N-termini length of 251 and 254, respectively.

### The *Glutamate* receptor family

The *Glutamate* family consists of 22 members in human. Most of these receptors have long N-termini that serve as an endogenous ligand binding region. Furthermore, the N-terminal region of the majority of receptors in human is also characterized with the presence of a cysteine rich domain (has 9 conserved cysteine residues) known as CRD or NCD3G. The ancient member of this family is present in species *T. pseudonana*.

#### Novel homologues

Significantly, 118 novel members of the *Glutamate* family were found, of which 96 receptors are found in Fungi, 1 in *Geodia cydonium* (Porifera), and 1 each in *M. brevicollis* and *S. rosetta* and 20 in *C. owczarzaki*. Surprisingly, 96 putative *Glutamate* receptors in Fungi are from only 4 species, with a remarkable number of 78 members in *A. macrogynus* from phylum Blastocladiomycota (the most found in any species including human), 14 in *S. punctatus* and 3 in *B. dendrobatidis* from phylum Chytridiomycota (which also have *Rhodopsin* and *Adhesion* receptor homologues) and 1 in *Rhizopus oryzae* from phylum Zygomycota. Similar to the previous reports, we did not find any *Glutamate* receptors in the Fungi phyla Ascomycota and Basidiomycota.

#### Conserved features

In the multiple sequence alignments there were partially or completely conserved residues across the 7TM region between the identified novel *Glutamate* family members and the characterized members of the *Glutamate* family in human (Figure S4). It is noteworthy that the 78 putative members of the *Glutamate* receptor family in *A. macrogynus* share about 27.4% identity in the 7TM regions and full length identity of just about 14.5%. This relatively low value suggests that even if these genes are a possible outcome of duplication, or local expansion, within the species, they seem to have diverged considerably.

#### Phylogenetic analysis

Phylogenetic analysis was performed including the novel members of the *Glutamate* family identified in Fungi, Choanoflagellata, Filasterea and Porifera with the known *Glutamate* receptors in human, *D. discoideum* and *T. pseudonana* (Figure S5). The analysis revealed that the putative *Glutamate* receptors identified in Fungi clustered (PP>80%) separately from the homologues of the *Glutamate* family in Metazoa. The sequences found in *C. owczarzaki* forms a distinct cluster with a posterior probability >70%. Further, the tree demonstrates that the sequence GB_CAA76688.1 in species *G. cydonium* from phylum Porifera cluster (PP 100%) with GABBR1 (gamma amino butyric acid B receptor) in human, which suggests a close relationship with each other.

#### N-terminal domain architecture

**Figure 5:**
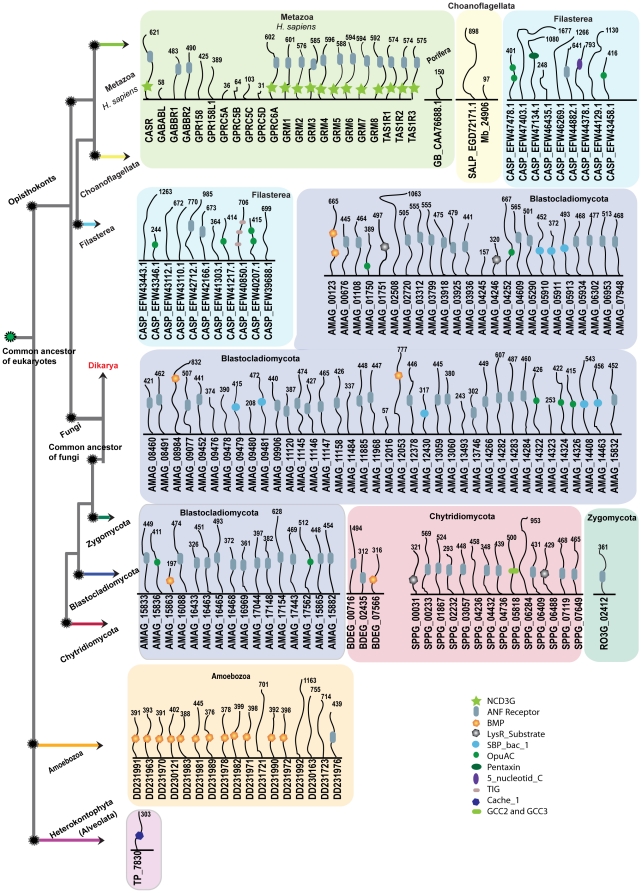
Schematic presentations of N-terminal domains of the Glutamate receptor family across the eukaryotic tree. The figure illustrates N-terminal domain architecture of the *Glutamate* receptors across different kingdoms of eukaryotes. The domains were identified with Pfam search and verified with RPS-Blast with a cutoff e-value of 0.1. For comparison, we represent known domain architecture of 22 *Glutamate* receptors from *Homo sapiens* for Metazoan lineage and 17 from *D. discoideum* for Amoebozoa and 1 from *T. pseudonana* for Alveolata. The illustration displays newly identified sequences from other eukaryotic lineages like Porifera (*G. cydonium*) a sister group to Metazoans, Choanoflagellata (*M. brevicollis* and *S. rosetta*), Filasterea (*C. owczarzaki*) and 4 genomes of Fungi; 1 for Blastocladiomycota (*A. macrogynus*) 2 representing for Chytridiomycota (*B. dendrobatidis, S. punctatus*) and 1 for Zygomycota (*R. oryzae*). The sequences are grouped respective to the major lineages across the eukaryotic tree. The colored boxes indicate the sequences to which phylum they belong to. The loss of *Glutamate* receptors in Dikarya which descended from a common ancestor of Fungi is indicated in red. The numbers at the top of each connective thread along domain symbols indicates the length of the N-termini. Each domain is marked with a symbol and abbreviated in the lower right corner. The domains are NCD3G (Nine Cysteines Domain of family 3 GPCR), ANF receptor (Receptor family ligand binding region), BMP (Basic membrane protein), LysR_Substrate (LysR substrate binding domain), SBP_bac_1 (Bacterial extracellular solute-binding protein), OpuAC (Substrate binding domain of ABC-type glycine betaine transport system), Pentaxin domain, 5_nucleotid_C domain, TIG (this family consists of a domain that has an immunoglobulin like fold), Cache_1 (Cache domain) and GCC2_GCC3.

The N-termini of almost all identified members of the *Glutamate* family in Fungi and other eukaryotic lineages, was very similar in both length and the nature of the functional domains present in the *Glutamate* family receptors in human (Figure 5). The length of the N-termini of the novel sequences range from 57–1667 (average of 497) residues, which was similar to humans, which range from 31–621 (average of 446) residues. The functional domains included the ANF_receptor (PF01094), which is one of the members of clan Periplasmic binding protein like (CL0144; Periplas_BP) and is part of the N-terminal region for CASR, GABBR1 &2, GRMs and TAS1Rs receptors in human. Curiously, the ANF_receptor domain was also present in most of the *Glutamate* family members identified in Fungi, 3 sequences in *C. owczarzaki* and also in sequence DD231976 identified in *D. discoideum*. It is noteworthy that GB_CAA76688.1 from the phylum Porifera, which clusters with GABBR1 in the phylogenetic tree, lacks the ANF_receptor domain. Further, additional domains were found that belong to the same Pfam clan CL0144; Periplas_BP. This includes BMP (PF02608) domain in 13 members of the *Glutamate* family identified in *D. discoideum* and in two sequences AMAG_08984, AMAG_12053 from *A. macrogynus*. In addition, we found other periplasmic binding protein domains that are (grouped in a separate Pfam clan, PBP: CL0177) not found in human *Glutamate* receptors, but found in putative *Glutamate* receptors identified in Fungi. They are LysR_substrate (PF03466) domain (in AMAG_01751, 04246, SPPG_00031), PBP_like_2 (PF12849) domain (in SPPG_06488), OpuAC (PF04069) domain (in AMAG_01750, 04252, 14324, 14326, 15836 and 17562) and SBP_bac_1 (PF01547) domain in 8 sequences in *A. macrogynus*. But the NCD3G (PF07562) domain found in CASR, GRMs, TAS1Rs in human is absent in all identified novel non-Metazoan members.

Further, domains were found that have not been reported previously in the *Glutamate* family like Pentaxin (PF00354) domain in CASP_EFW47134.1, 5_nucleotid_C (PF02872) domain in CASP_EFW44378.1, TIG (PF01833) domain in CASP_EFW40850.1 from *C. owczarzaki* and Cache_1 (PF02743) domain in TP_7830 in *T. pseudonana* from Alveolata. It is noteworthy that the Pentaxin domain is found in the *Adhesion* family receptors GPR144 and GPR126 in humans and TIG domain in the *Adhesion* receptors from *S. rosetta*.

### The *Frizzled* receptor family

The *Frizzled* family of GPCRs consists of 10 receptors (FZD1–10) in human [10]. They are mostly known as receptors for Wnt proteins and play key role in tissue polarity and cell signaling [1]. The family members are characterized by the CRD_FZ domain or FZ domain which has 10 conserved cysteine residues. The ancient member or origin of this family can be traced back to *D. discoideum* [8].

#### Novel homologues

Our analysis identified 6 new *Frizzled* receptor sequences, distributed as 2 in Fungi (*S. punctatus*) and 4 in sponges (3 sequences in species *Amphimedon queenslandica*, 1 in *Suberites domuncula*), which belong to phylum Porifera. However, we did not find any *Frizzled* receptors in Choanoflagellata, Filasterea, Fungi phyla Ascomycota and Basidiomycota and in Alveolata.

#### Conserved features

Multiple sequence alignment of the known *Frizzled* receptors in human and 6 novel sequences in Fungi and Porifera revealed that they are conserved with an identity of about 40%. Multiple cysteine residues along the sequence of the frizzled receptors were found to be completely conserved in most, and partially in a few cases (Figure S6).

#### Phylogenetic analysis

Phylogenetic analysis of the *Frizzled* family was performed using the sequences from human, *N. vectensis*, *T. adhaerens* and *D. discoideum* and compared to the novel homologues from Fungi and Porifera (Figure S7). The tree is rooted with the node clustering the ancient members of the family in *D. discoideum*. The analysis revealed that the two sequences SPPG_01720 and SPPG_06172 in *S. punctatus* of Fungi cluster together (PP 100%) and placed basal to the node which cluster (PP 100%, 492/500) the Metazoan homologues of the *Frizzled* family. Furthermore, the tree demonstrates that the two sequences ADO16580.1 and ADO16569.1 from phylum Porifera (sponges), the closest relatives or sister group to the Metazoans do not show orthologous relationship with Metazoan receptors. In contrast, the other two sequences ADO16570.1 and CAD97575.1 identified in Porifera, cluster (PP 98%) together along with the sequences FZD4, FZD9 and FZD10 from humans, TA_31674 from *T. adhaerens*, NV_168924 and NV_139208 from *N. vectensis*.

### Fungi specific GPCRs

In this study, we also searched for the previously known Fungi kingdom specific GPCRs that involve in pheromone sensing (Ste2, Ste3), sensors for glucose (Git3), and nitrogen sensors (stm1) among others. We identified 619 fungal specific GPCRs across the 79 species analyzed in this study (Table S3). Surprisingly, we could not find reliable homologues for Ste2, Ste3 and Git3 in Phylum Chytridiomycota that instead has homologues for mammalian like GRAFS families. Similarly, Zygomycetes *R. oryzae* has no homologues except for the nutrient sensing receptors. This observation is in line with the previous report on *R. oryzae* [18]. It is noteworthy that a widespread representation of these unique Fungi specific GPCRs was found only in Ascomycota and Basidiomycota that thoroughly lack the mammalian like GRAFS families.

## Discussion

**Figure 6:**
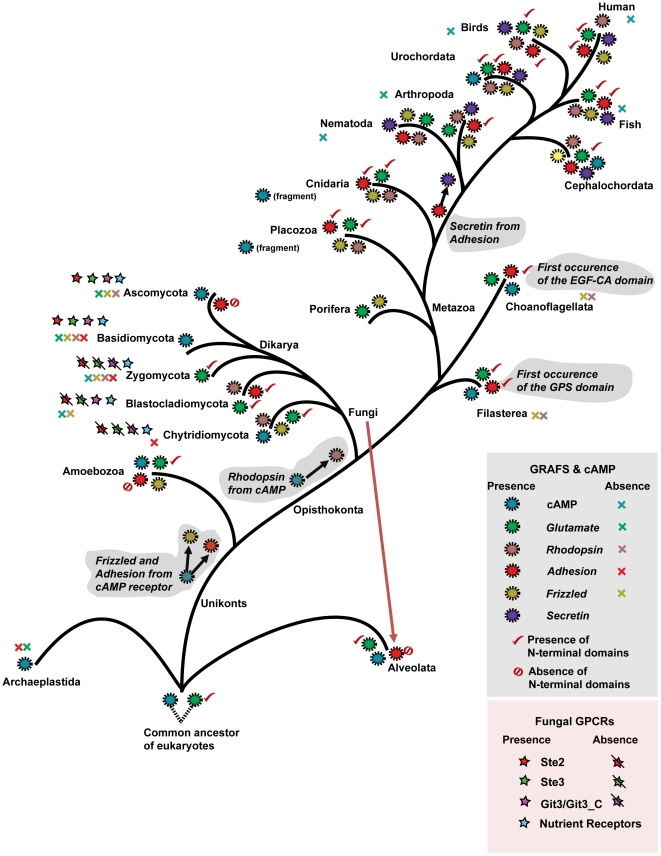
Schematic presentation of the origin, evolution and lineage-specific losses of the five main GRAFS families, cAMP receptor family and the Fungi kingdom specific GPCRs. The eukaryotic evolutionary tree is constructed with references from the tree of life Web project (http://tolweb.org/tree/phylogeny.html). Each branch shows the presence (colored circles) and the loss (colored cross symbol) of the five main GRAFS families and the cAMP receptor family. The presence and absence of the N-terminal domains of the *Glutamate* and the *Adhesion* family is indicated with a tick mark and a crossed circle, respectively. The presence of Fungi kingdom specific GPCRs were represented by colored star symbols, and their absence with a line segment in black against the respective colored star symbol. The putative connection and origin of the *Glutamate* and cAMP receptor family is indicated with dotted lines at the bottom. The horizontal gene transfer of the *Adhesion* receptor family from Fungi to Alveolata is indicated with dotted lines in red. Branch lengths are not drawn to represent actual evolutionary distances.

Here we provide the first evidence that four of the five main mammalian families of GPCRs, namely *Rhodopsin*, *Adhesion*, *Glutamate* and *Frizzled*, are present in Fungi. The evidence is convincing for all the families and we were able to create alignments of the human and fungal members for each of the families. The results are further supported by a number of motifs that seem remarkably conserved and these are likely to be crucial for the function of the family specific properties of these receptors. Also, we identified GPCRs in *T. pseudonana* and *P. tetraurelia*, which belong to the eukaryotic kingdom known as Chromalveolata. Based on this comprehensive study covering 83 species, we establish that the origin of the *Rhodopsin* family could be traced back to the ancestor of Opisthokonts (∼1100 MYA), the *Adhesion* and the *Frizzled* family to Unikonts (∼1275 MYA) and the *Glutamate* and the cAMP receptor family to the common ancestor of Alveolates and Unikonts, early in eukaryotic evolution (>1400 MYA) (Figure 6). We provide higher resolution and deduced precise timeframe of the origin and diversification of the GRAFS families than previous reports [8]. We establish that the *Frizzled* and the *Adhesion* family evolved from the cAMP receptor family before the split of Unikonts from the common ancestor of eukaryotes. The *Rhodopsin* family evolved from the cAMP receptor family in the common ancestor of Opisthokonts, after the divergence of *D. discoideum*. It is interesting to note the similarities between the cAMP and *Rhodopsin* families, suggesting that the *Rhodopsin* family is an expansion of the more ancient cAMP branch perhaps taking over its functions in more complex organisms while cAMP receptors then become redundant. Later, the *Secretin* family evolved from the *Adhesion* family in an event that happened between the split of Cnidaria (*N. vectensis*) and the split of Nematoda (*C. elegans*) in the Metazoan lineage, as we previously reported [14]. Also, we have traced the lineage and species specific losses of these families across the eukaryotic tree (Figure 6). This study also highlights the importance of an ancient role of the N-terminal functional domains of the *Adhesion* and the *Glutamate* families across the diverse eukaryotic lineages. Intriguingly, the widespread distribution of these classical membrane receptors across the eukaryotic domain demonstrates that evolutionary divergent eukaryotes like the unicellular Alveolates and the complex multicellular organisms of Metazoan lineage share a basal signal transduction system that was present already in early eukaryotic evolution.

### *Rhodopsin* and cAMP receptor family

We provide convincing evidence that the split of the large *Rhodopsin* family from the cAMP receptor family can be placed at the emergence of the common ancestor of Opisthokonts (Fungi and Metazoa) and not in Placozoa (basal lineage of Metazoa) as earlier assumed [8]. Interestingly, we found that the newly identified sequences of the *Rhodopsin* and the cAMP receptor family in the basal eukaryotic genomes share conserved motifs between them. This includes the important DRY and NPXXY motifs of the *Rhodopsin* family that are crucial to the functional integrity of these receptors [25], [26]. These motifs are present as ERY and NPXXX in the 12 novel *Rhodopsin* family sequences in Fungi and N(S)XY and NS(A)XXY in the cAMP receptors from Fungi and Alveolata, respectively (Figure 1). This conservation within the 7TM region strongly links these families. In addition, the phylogenetic tree clearly clusters (PP>90%, 466/500) all the novel sequences from the *Rhodopsin* family and cAMP receptor family into two separate groups and suggest a common origin between these two families (Figure 2). Also, the scatter plot (Figure 3) clearly demonstrates the similarity between the Pfam HMM models of the *Rhodopsin* and the cAMP receptor family. This is in good agreement with our earlier report that the *Rhodopsin* family and the cAMP receptor family share an HHsearch (HMM-HMM search) homology probability of 99.4% [8]. Also, we noticed insensitivity of the Pfam HMM models to distinguish between the two families prevails right at the point where the split of the *Rhodopsin* family from cAMP receptor family have occurred. Further, the cAMP receptor family is present in plants, Alveolata, Amoebozoa and Fungi but subsequently become redundant in the Metazoan lineage as it is found in 3 invertebrate species and lost in the vertebrates (Figure 6). Taken all these findings together, it is evident that the cAMP receptor family gave rise to the *Rhodopsin* family in an event close to the common ancestor of Opisthokonts and then persisted and evolved as a large *Rhodopsin* family in the Metazoan lineage.

### *Adhesion* receptor family

We have traced the origin of the *Adhesion* family to the ancestor of Unikonts (Figure 6). Here we provide the first evidence for the presence of *Adhesion* receptors in Filasterea (*C. owczarzaki*), Choanoflagellata (*S. rosetta*) and in two of the four major phyla of the Fungi kingdom. Also, our search emphasize that *Adhesions* are not found in eukaryotic lineages before the split of *D. discoideum*. The sole representative of *Adhesions* from Alveolata is a possible horizontal gene transfer from Fungi, since it cluster with the *Adhesion* receptors found in Ascomycota of Fungi (PP 99%) (Figure S3). This is also supported by a pair wise identity of about 50% in the 7TM region (Figure S8), which is relatively high for lineages that are as distant as Fungi and Alveolata. Considering these findings together with our earlier report showing an HHsearch homology probability of 99.8% with the cAMP receptor family [8], we validate that the *Adhesion*s evolved from the ancient cAMP receptor family in an event close to the split of Unikonts from the common ancestor of eukaryotes.

Intriguingly, the complexity of the *Adhesion* receptors with long N-termini containing multiple functional domains observed in the Metazoan lineage is absent in receptors from Fungi, Amoebozoa and Alveolata, whereas the 7TM region is conserved in species ranging from unicellular Alveolates to human (Figure 4). These results may suggest that ligand binding and signaling mechanisms of the *Adhesion* receptors in Fungi and other basal eukaryotes could be confined within the 7TM region. However, the conservation of these motifs in the Metazoan lineage suggests that they are important for the signaling mechanisms along with the long N-termini with multiple functional domains. The complex multi-domain N-termini that emerged close to the radiation and diverged within the Metazoan lineage might reflect the need for a broader signal transduction system in the intricate cell to cell communication that is characteristic for Metazoan multicellularity. Prime examples are the emergence of the characteristic *Adhesion* family domains, GPS and Calx-beta domain in Filasterea and EGF-CA domain in Choanoflagellata, which prevailed in the multicellular Metazoan species (Figure 6). In addition, we extended the knowledge of the N-terminal region of the *Adhesions* by adding at least three novel domains TIG, TLD and RLD to the known 31 unique domains [14].

### *Glutamate* receptor family

Our results suggest that the origin of the ancient *Glutamate* receptor lies in the common ancestor of eukaryotes, since we found them in Chromalveolata, Unikonts and Opisthokonts. For the first time, we have found 120 novel *Glutamate* receptors in the basal Opisthokont lineages which include Fungi and the close relatives of animals- Choanoflagellata, Filasterea and Porifera. We have also shown that they are also present in *D. discoideum* and *T. pseudonana*, which diverged before the Opisthokont lineage [8]. We found that the ancient *Glutamate* family is present in almost all eukaryotic lineages except in plants and in the Fungi subkingdom Dikarya, which include Ascomycota and Basidiomycota (see Figure 6).

Interestingly, the conserved length and the nature of the functional domains within the N-termini across all eukaryotic lineages in which the *Glutamate* receptors were found suggest that the endogenous ligand binding or so called Venus flytrap mechanism (VFTM) and the subsequent signal transduction mechanism emerged early in the eukaryotic life (Figure 5). This is in contrast with the *Adhesion* receptor family where the N-termini complexity is absent in the basal eukaryotes. Further, we found that >90% of the functional domains in the N-termini of the *Glutamate* receptors in diverse eukaryotic lineages, belong to the periplasmic binding protein superfamily. This includes the domain ANF Receptor and the four novel domains, BMP, LysR_Substrate, SBP_bac_1 and OpuAC that have not previously been identified in the *Glutamate* receptors. Taken together, it suggests a conserved fundamental role and remarkable adaptability of these periplasmic binding protein superfamily domains in regulating the signaling mechanisms of the *Glutamate* receptors from unicellular organisms to human.

### *Frizzled* receptor family

We establish that the *Frizzled* family evolved from the cAMP receptor family before the divergence of Unikonts from a common ancestor of eukaryotes. This is supported by our extensive search in genomes that diverged before the eukaryotic supergroup Unikonts, which showed that they are absent in plant kingdom and Alveolata. Our earlier report showed that the *Frizzled* share an HHsearch homology probability of 99.4% with the cAMP receptor family [8]. However, we cannot trace which of the *Adhesion* and the *Frizzled* family diverged first; as they evolved within the same timeframe from the cAMP receptor family (see Figure 6). In addition, here we provided the first identification of 6 novel *Frizzled* family receptors in Fungi and Porifera. Interestingly, they are present only in the basal fungal lineage Chytridiomycota, which also have *Rhodopsin*, *Adhesion* and *Glutamate* families that all together account to 112 novel receptors. This suggests that Chytridiomycota phylum have remarkable necessity for a broader signaling system than the other 3 major phyla of Fungi that resembles the Metazoan GPCR repertoire.

### Fungi specific GPCRs

Our results suggest that the pheromone sensing receptors (Ste2, Ste3) that are mainly involved in exchanging mating signals across the individuals of the same species, emerged after the split of Dikarya subkingdom. Interestingly, the Chytridiomycota has only Metazoan GPCRs (except the presence of Fungi nutrient sensing GPCRs) while Dikarya, which evolved later, has only fungal specific GPCRs. This could suggest that the basal fungal lineages that had the Metazoan like GPCR signaling system could have evolved later to become fungal specific GPCRs in Ascomycota and Basidiomycota. This observation finds support from our previous report which suggests that these pheromone sensing GPCRs were closely related to cAMP receptors with an HHsearch homology probability of 96.1% [8]. Considering the fact that the *Dictyostelium* cAMP receptors are involved in sexual chemotaxis and development [27], [28], it is thus tempting to speculate that *Dictyostelium* cAMP like receptors in Chytridiomycota may perform similar roles in finding mates and that these functions could be analogous to the function of pheromone receptors in Dikarya.

### Novel mammalian like GPCRs: a possible antifungal drug targets and candidates for deorphanization?

GPCRs mediated signaling is known to be involved in fungal pathogenic morphogenesis and mycotoxin production in filamentous Fungi [22], [29]. Despite the role of GPCRs in fungal pathogenesis, there is no current development of potential antifungal drugs that target GPCRs. There are several recent reports of infectious diseases caused by Fungi that affect humans and wild life [30], [31]. To highlight a few, *Cryptococcus gattii* (Basidiomycota) causes infectious diseases in immunocompromised individuals in United States and *B. dendrobatidis* that belong to the group Rhizophydiales under the phylum Chytridiomycota is a major cause for the decline of amphibians [32], [33]. As novel fungal pathogens are emerging, there is an increasing need to develop novel drugs in this field. One of the possible reasons for the lack of development of the drugs is there are only few fungal GPCRs known and these are not strongly related to the well characterized mammalian like GPCR families that are major drug targets [1], [34]. Here, we have identified the first mammalian like GPCRs in the basal fungal phyla Chytridiomycota and Blastocladiomycota, creating an opportunity to delineate potential antifungal targets and aid deorphanization of these receptors by comparison with the well studied mammalian counterparts that serve as targets for 36% of the available drugs.

## Materials and Methods

### Genomes and proteomes

Complete proteome and genome dataset for 79 fungal species were downloaded. The dataset consists of proteomes for 61 species from UniProt (http://www.uniprot.org), which are distributed in phylum Ascomycota (48 species), Basidiomycota (9 species), Microsporidia (4 species). Other 18 Fungi proteomes were downloaded from FGI endorsed by Broad institute at MIT (http://www.broad.mit.edu/annotation/fgi), which are distributed in Ascomycota (13 species), Basidiomycota (1 species), Zygomycota (1 species) and in Chytridiomycota (3 species). In addition to the fungal proteome dataset, the study investigated proteomes for unicellular species with key evolutionary positions within the eukaryotic tree: *S. rosetta* [35], *C. owczarzaki* [36], *P. tetraurelia* [37] from NCBI (http://www.ncbi.nlm.nih.gov/), and *T. thermophila* [38] from UniProt. Furthermore, a collection of proteins (3465 proteins from several species) were obtained from NCBI that represents the phylum Porifera, a basal group of animals [39].

### Sequence retrieval, mining and removal

*A) GRAFS families*: Full-length *Rhodopsin*, *Adhesion*, *Glutamate* and *Frizzled* family sequences for representative Metazoan genomes and the cAMP receptor family sequences from *D. discoideum* and *C. intestinalis* were retrieved from previously published GPCR repertoires to compare the common motifs of individual families and to perform phylogenetic analysis [3]–[8]. GPCRs in 79 fungal and the remaining genomes from other eukaryotic lineages were mined using HMM searches performed using the recent version of profile HMM software HMMER3 (http://hmmer.janelia.org/) with the Pfam_scan.pl script available at the Pfam homepage [40]. The Pfam_scan.pl script aligns sequences with the HMMs corresponding to the Pfam domains and only keeps the best aligned Pfam domain for each region. The putative dataset was aligned to the complete Pfam database version 24, which has 11912 families with sensitive HMM models built using HMMER3 software [41]. For the search against the complete Pfam database, the standard settings were utilized as provided by Pfam_scan.pl. We retrieved only the sequences which received the Pfam domains 7tm_1 (PF00001), 7tm_2 (PF00002) 7tm_3 (PF00003), *Frizzled* (PF01534) and Dicty_CAR (PF05462) from each proteome and classified them into GPCR families based on the Pfam result. The sequences with a 7TM domain predicted to have fewer than six or more than nine transmembrane segments in Phobius [42] were excluded from further analysis in order to reduce the number of incomplete sequences. Furthermore, the program CD-HIT [43] was run within the sequences of each genome separately with 90% cutoff to reduce the size of the dataset.

*B) Fungi specific GPCRs*: Similar to the methodology mentioned above, we retrieved the sequences that received the Pfam domains STE2 (PF02116), STE3 (PF02076), Git3 (PF11710)/Git3_c (PF11970). For nutrient receptors, we constructed an HMM profile from the sequences retrieved from [18]. The HMM profile for the nutrient receptors was then used as a query for the HMMSEARCH program (with strict e-value cutoff of 1e^−25^) to search against our complete proteome dataset analyzed in this study. We identified 619 Fungi specific GPCRs from the 79 fungal genomes analyzed in this study. The accession numbers for all the identified sequences were given in Table S3.

### Alignments and phylogenetic analysis

The selected GPCR candidates were aligned using MAFFT version 6 (MAFFT, (http://mafft.cbrc.jp/alignment/server/) using the E-INS_I version (optimal for sequences with conserved motifs and carrying multiple domains) with default parameters [44]. All the alignments and the phylogenetic analyses were constructed based only on the seven transmembrane spanning regions and, for the phylogenetic analysis, unaligned regions were removed. Consensus sequences for each of the subgroups reported in Figure 1 were generated from all available human GPCR sequences for each of the subfamilies. The sequences were obtained from our human GPCR repertoire [4]. Similarly, a consensus sequence for the cAMP receptor family homologues in Fungi (Figure 1) were generated from the identified sequences in this study. First, the sequences that belong to each of the subgroups were aligned and separate HMM profiles were built from those alignments. Each HMM profiles that are respective to the groups serves as an input for the HMMEMIT program and a consensus sequence were obtained using option “-C” as implemented in the HMMER3 package. The consensus sequence is formed using a plurality rule that selects the maximum probability residue at each match state from the HMM profiles. The alignments were inspected and edited using Jalview. The phylogenetic analysis was performed using a Bayesian approach as implemented in MrBayes version 3.1.2 [45]. Markov Chain Monte Carlo (MCMC) analysis was used to approximate the posterior probabilities of the trees. Analysis was run using a gamma shaped model for the variation of evolutionary rates across sites (rates = gamma) and the mixed option (aamodelpr = mixed) was used to estimate the best amino acid substitution model. Each analysis was set to run for 3000000 generations and every hundredth tree was sampled. A stop rule was applied to determine when to terminate the MCMC generations as recommended in the MrBayes manual (standard deviation of split frequencies <0.01). If the MCMC analysis doesn't hit the stop value within the default number of generations, additional generations were run for it to reach the minimum. The first 25% of the sampled trees were discarded (burnin = 0.25) to reassure a good sample from the posterior probability distribution. A consensus tree was built from the remaining 75% of the sampled trees with the MrBayes *sumt* command using the 50% majority rule method. The *sump* command was used to control so that an adequate sample of the posterior probability distribution was reached during the MCMC procedure. The topology of all the phylogenetic trees supported by the posterior probability (PP) of the Bayesian approach was cross verified with bootstrap analysis (500 replicates) using maximum likelihood (ML) approach as implemented in PhyML (version 3.0) program [46]. Bootstrap values were indicated for the nodes that received good support in ML approach. The phylogenetic tree was drawn in FigTree 1.3.1 (http://tree.bio.ed.ac.uk/software/figtree/).

### Domain search

The N-terminal domains for the identified *Adhesion* and *Glutamat*e family members were identified with Pfam search and also verified using RPS-blast with a cutoff e-value of 0.01 against the Conserved Domain Database (CDD) version 2.29 position-specific scoring matrixes (PSSMs).

## Supporting Information

Dataset S1 Amino acid sequences of all novel sequences identified in this study in standard FASTA format.
(TXT)

Table S1 Accession numbers of novel sequences identified in this study.
(XLS)

Table S2 List of species investigated.
(DOC)

Table S3 Accession numbers of all Fungi specific GPCRs identified in this study.
(XLS)

Figure S1 Phylogenetic relationship between the *Rhodopsin* family sequences in Fungi and human.
(TIF)

Figure S2 Phylogenetic relationship between the *Rhodopsin* family sequences in Fungi and *T. adhaerens*.
(TIF)

Figure S3 Phylogenetic relationship between the novel *Adhesion* family sequences in Fungi, Filasterea, Choanoflagellata and Alveolata with the representatives from Metazoa. The tree is rooted with *D. discoideum*. The node that is highlighted in red clustered the *Adhesion* receptor from Alveolata with the fungal members (PP>90%). They share about 50% identity within the 7TM regions (see Figure S8).
(TIF)

Figure S4 Alignment of the *Glutamate* family sequences in diverse eukaryotic lineages. The alignment shows the conserved regions within the 7TM region between the novel sequences in diverse eukaryotic lineages and human. The consensus sequence for each species was obtained from separate alignments. The number of sequences aligned to emit the consensus sequences for each species is given in the parenthesis. Regions which show >50% conservation are highlighted.
(TIF)

Figure S5 Phylogenetic relationship between the *Glutamate* family sequences in Fungi, Choanoflagellata, Porifera and Alveolata with human.
(TIF)

Figure S6 Alignment of the novel *Frizzled* receptor sequences in Fungi and Porifera with the representative consensus sequences of the *Frizzled* receptors from human, *N. vectensis* (NV), *T. adhaerens* (TA) and *D. discoideum* (dicty). Regions which show >50% conservation are highlighted. Multiple cysteine residues that are characteristic for the *Frizzled* family are mostly conserved in the novel sequences.
(TIF)

Figure S7 Phylogenetic relationship between the *Frizzled* family sequences in Fungi and sponges (Porifera) with the representatives from Metazoa.
(TIF)

Figure S8 Alignment showing the conservation in the 7TM region between the *Adhesion* receptor sequences in Fungi and Alveolata. Regions which show more than 50% conservation are highlighted.
(TIF)
