# Supplementary material for: The Origin of GPCRs: Identification of Mammalian like Rhodopsin, Adhesion, Glutamate and Frizzled GPCRs in Fungi
Source: PLoS One. 2012 Jan 4;7(1):e29817. doi: 10.1371/journal.pone.0029817 (PMC3251606; doi:10.1371/journal.pone.0029817)
Supplement: Table S2 — List of species investigated. (DOC) [file pone.0029817.s003.doc]

**Table S2.** List of species investigated.

| **Kingdom/Phylum** | **Species** | **References** |
| --- | --- | --- |
| **Fungi** |  |  |
| Ascomycota | *Ajellomyces capsulata* | U |
|  | *Ajellomyces dermatitidis* | U |
|  | *Arthroderma otae* | U |
|  | *Ashbya gossypii* | [1] |
|  | *Aspergillus clavatus* | U |
|  | *Aspergillus flavus* | U |
|  | *Aspergillus fumigatus* | [2] |
|  | *Aspergillus niger* | [3] |
|  | *Aspergillus oryzae* | [4, 5] |
|  | *Aspergillus terreus* | U |
|  | *Botryotinia fuckeliana* | [6] |
|  | *Candida albicans* | [7] |
|  | *Candida dubliniensis* | [8] |
|  | *Candida glabrata* | U |
|  | *Candida guillermondii* | B (FGI) |
|  | *Candida tropicalis* | U |
|  | *Chaetomium globosum* | U |
|  | *Clavispora lusitaniae* | U |
|  | *Coccidioides immitis* | [9] B (FGI) |
|  | *Coccidioides posadasii* | [9] |
|  | *Colletotrichum graminicola* | U |
|  | *Debaryomyces hansenii* | U |
|  | *Emericella nidulans* | [10] |
|  | *Fusarium graminearum* | [11] B (FGI) |
|  | *Fusarium oxysporum* | [11] B (FGI) |
|  | *Fusarium verticillioides* | [11] B (FGI) |
|  | *Kluyveromyces lactis* | [12] |
|  | *Lachancea thermotolerans* | [13] |
|  | *Magnaporthe grisea* | [14] B (FGI) |
|  | *Magnaporthe oryzae* | U |
|  | *Microsporum gypseum* | # B (FGI) |
|  | *Nectria haematococca* | [15] |
|  | *Neosartorya fischeri* | U |
|  | *Neurospora crassa* | [16] |
|  | *Penicillium chrysogenum* | [17] |
|  | *Penicillium marneffei* | [18] |
|  | *Phaeosphaeria nodorum* | [19] |
|  | *Pichia pastoris* | [20] |
|  | *Pichia stipitis* | [21] |
|  | *Podospora anserina* | [22] |
|  | *Pyrenophora teres f. teres* | [23] |
|  | *Pyrenophora tritici-repentis* | U |
|  | *Saccharomyces cerevisiae* | [24, 25]] |
|  | *Schizosaccharomuyces octosporus* | [26] |
|  | *Schizosaccharomyces pombe* | U |
|  | *Sclerotinia sclerotiorum* | [27] |
|  | *Sordaria macrospora* | [28] |
|  | *Stagonospora nodorum* | [19] B (FGI) |
|  | *Talaromyces stipitatus* | U |
|  | *Trichophyton equinum* | B (FGI) |
|  | *Trichophyton rubrum* | B (FGI) |
|  | *Trichophyton tonsurans* | B (FGI) |
|  | *Uncinocarpus reesii* | U |
|  | *Vanderwaltozyma polyspora* | [29] |
|  | *Verticillium albo-atrum* | U |
|  | *Verticillium dahliae #* | [30] B (FGI) |
|  | *Yarrowia lipolytica* | U |
|  | *Zygosaccharomyces rouxii* | U |
| Basidiomycota | *Coprinopsis cinerea* | [31] |
|  | *Coprinus cinereus* | B (FGI) |
|  | *Cryptococcus neoformans* | [32] |
|  | *Laccaria bicolor* | [33] |
|  | *Malassezia globosa* | [34] |
|  | *Moniliophthora perniciosa* | U |
|  | *Postia placenta* | [35] |
|  | *Puccinia graminis f. sp. tritici* | U |
|  | *Schizophyllum commune* | U |
|  | *Ustilago maydis* | [36] |
| Microspordia | *Encephalitozoon cuniculi* | [37] |
|  | *Encephalitozoon intestinalis* | [38] |
|  | *Enterocytozoon bieneusi* | [39] |
|  | *Nosema ceranae* | U |
| Zygomycota | *Rhizopus oryzae* | [40] B (FGI) |
| Blastocladiomycota | *Allomyces macrogynus* | B (FGI) |
| Chytridiomycota | *Batrachochytrium dendrobatidis* | B (FGI) |
|  | *Spizellomyces punctatus* | B (FGI) |
| **Filasterea** | *Salpingocea rosetta* | B (ORG) |
| **Choanoflagellata** | *Capsaspora owczarzaki* | B (ORG) |
| **Alveolata** | *Paramecium tetraurelia* | [41] B (ORG) |
|  | *Tetrahymena thermophila* | [42] B (ORG) |

**Note:** U- Proteomes downloaded from UniProt (<http://www.uniprot.org/>)

B (FGI) - Proteomes downloaded from FGI, Fungal genome initiative (<http://www.broadinstitute.org/scientific-community/science/projects/fungal-genome-initiative/fungal-genome-initiative> ) endorsed by Broad institute at MIT ([http://www.broadinstitute.org](http://www.broadinstitute.org/))

B (ORG) - Proteomes downloaded from Origins of Multicellularity Database (<http://www.broadinstitute.org/annotation/genome/multicellularity_project/MultiHome.html> ) endorsed by Broad institute at MIT ([http://www.broadinstitute.org](http://www.broadinstitute.org/)).

*The complete proteomes of the species that are published as an article were given in the reference list. For the rest, the database from which the proteomes were downloaded is acknowledged.*

**References:**

1. Dietrich FS, Voegeli S, Brachat S, Lerch A, Gates K, et al. (2004) The Ashbya gossypii genome as a tool for mapping the ancient Saccharomyces cerevisiae genome. Science 304: 304-307.

2. Nierman WC, Pain A, Anderson MJ, Wortman JR, Kim HS, et al. (2005) Genomic sequence of the pathogenic and allergenic filamentous fungus Aspergillus fumigatus. Nature 438: 1151-1156.

3. Pel HJ, de Winde JH, Archer DB, Dyer PS, Hofmann G, et al. (2007) Genome sequencing and analysis of the versatile cell factory Aspergillus niger CBS 513.88. Nat Biotechnol 25: 221-231.

4. Galagan JE, Calvo SE, Cuomo C, Ma LJ, Wortman JR, et al. (2005) Sequencing of Aspergillus nidulans and comparative analysis with A. fumigatus and A. oryzae. Nature 438: 1105-1115.

5. Machida M, Asai K, Sano M, Tanaka T, Kumagai T, et al. (2005) Genome sequencing and analysis of Aspergillus oryzae. Nature 438: 1157-1161.

6. Catlett NL, Yoder OC, Turgeon BG (2003) Whole-genome analysis of two-component signal transduction genes in fungal pathogens. Eukaryot Cell 2: 1151-1161.

7. Jones T, Federspiel NA, Chibana H, Dungan J, Kalman S, et al. (2004) The diploid genome sequence of Candida albicans. Proc Natl Acad Sci U S A 101: 7329-7334.

8. Jackson AP, Gamble JA, Yeomans T, Moran GP, Saunders D, et al. (2009) Comparative genomics of the fungal pathogens Candida dubliniensis and Candida albicans. Genome Res 19: 2231-2244.

9. Neafsey DE, Barker BM, Sharpton TJ, Stajich JE, Park DJ, et al. (2010) Population genomic sequencing of Coccidioides fungi reveals recent hybridization and transposon control. Genome Res 20: 938-946.

10. Prade RA, Griffith J, Kochut K, Arnold J, Timberlake WE (1997) In vitro reconstruction of the Aspergillus (= Emericella) nidulans genome. Proc Natl Acad Sci U S A 94: 14564-14569.

11. Ma LJ, van der Does HC, Borkovich KA, Coleman JJ, Daboussi MJ, et al. (2010) Comparative genomics reveals mobile pathogenicity chromosomes in Fusarium. Nature 464: 367-373.

12. Dujon B, Sherman D, Fischer G, Durrens P, Casaregola S, et al. (2004) Genome evolution in yeasts. Nature 430: 35-44.

13. Souciet JL, Dujon B, Gaillardin C, Johnston M, Baret PV, et al. (2009) Comparative genomics of protoploid Saccharomycetaceae. Genome Res 19: 1696-1709.

14. Dean RA, Talbot NJ, Ebbole DJ, Farman ML, Mitchell TK, et al. (2005) The genome sequence of the rice blast fungus Magnaporthe grisea. Nature 434: 980-986.

15. Coleman JJ, Rounsley SD, Rodriguez-Carres M, Kuo A, Wasmann CC, et al. (2009) The genome of Nectria haematococca: contribution of supernumerary chromosomes to gene expansion. PLoS Genet 5: e1000618.

16. Galagan JE, Calvo SE, Borkovich KA, Selker EU, Read ND, et al. (2003) The genome sequence of the filamentous fungus Neurospora crassa. Nature 422: 859-868.

17. van den Berg MA, Albang R, Albermann K, Badger JH, Daran JM, et al. (2008) Genome sequencing and analysis of the filamentous fungus Penicillium chrysogenum. Nat Biotechnol 26: 1161-1168.

18. Yuen KY, Pascal G, Wong SS, Glaser P, Woo PC, et al. (2003) Exploring the Penicillium marneffei genome. Arch Microbiol 179: 339-353.

19. Hane JK, Lowe RG, Solomon PS, Tan KC, Schoch CL, et al. (2007) Dothideomycete plant interactions illuminated by genome sequencing and EST analysis of the wheat pathogen Stagonospora nodorum. Plant Cell 19: 3347-3368.

20. De Schutter K, Lin YC, Tiels P, Van Hecke A, Glinka S, et al. (2009) Genome sequence of the recombinant protein production host Pichia pastoris. Nat Biotechnol 27: 561-566.

21. Jeffries TW, Grigoriev IV, Grimwood J, Laplaza JM, Aerts A, et al. (2007) Genome sequence of the lignocellulose-bioconverting and xylose-fermenting yeast Pichia stipitis. Nat Biotechnol 25: 319-326.

22. Paoletti M, Saupe SJ (2008) The genome sequence of Podospora anserina, a classic model fungus. Genome Biol 9: 223.

23. Ellwood SR, Liu Z, Syme RA, Lai Z, Hane JK, et al. (2010) A first genome assembly of the barley fungal pathogen Pyrenophora teres f. teres. Genome Biol 11: R109.

24. Goffeau A, Barrell BG, Bussey H, Davis RW, Dujon B, et al. (1996) Life with 6000 genes. Science 274: 546, 563-547.

25. Wei W, McCusker JH, Hyman RW, Jones T, Ning Y, et al. (2007) Genome sequencing and comparative analysis of Saccharomyces cerevisiae strain YJM789. Proc Natl Acad Sci U S A 104: 12825-12830.

26. Rhind N, Chen Z, Yassour M, Thompson DA, Haas BJ, et al. (2011) Comparative functional genomics of the fission yeasts. Science 332: 930-936.

27. Amselem J, Cuomo CA, van Kan JA, Viaud M, Benito EP, et al. (2011) Genomic analysis of the necrotrophic fungal pathogens Sclerotinia sclerotiorum and Botrytis cinerea. PLoS Genet 7: e1002230.

28. Nowrousian M, Stajich JE, Chu M, Engh I, Espagne E, et al. (2010) De novo assembly of a 40 Mb eukaryotic genome from short sequence reads: Sordaria macrospora, a model organism for fungal morphogenesis. PLoS Genet 6: e1000891.

29. Scannell DR, Frank AC, Conant GC, Byrne KP, Woolfit M, et al. (2007) Independent sorting-out of thousands of duplicated gene pairs in two yeast species descended from a whole-genome duplication. Proc Natl Acad Sci U S A 104: 8397-8402.

30. Klosterman SJ, Subbarao KV, Kang S, Veronese P, Gold SE, et al. (2011) Comparative genomics yields insights into niche adaptation of plant vascular wilt pathogens. PLoS Pathog 7: e1002137.

31. Stajich JE, Wilke SK, Ahren D, Au CH, Birren BW, et al. (2010) Insights into evolution of multicellular fungi from the assembled chromosomes of the mushroom Coprinopsis cinerea (Coprinus cinereus). Proc Natl Acad Sci U S A 107: 11889-11894.

32. Loftus BJ, Fung E, Roncaglia P, Rowley D, Amedeo P, et al. (2005) The genome of the basidiomycetous yeast and human pathogen Cryptococcus neoformans. Science 307: 1321-1324.

33. Martin F, Aerts A, Ahren D, Brun A, Danchin EG, et al. (2008) The genome of Laccaria bicolor provides insights into mycorrhizal symbiosis. Nature 452: 88-92.

34. Xu J, Saunders CW, Hu P, Grant RA, Boekhout T, et al. (2007) Dandruff-associated Malassezia genomes reveal convergent and divergent virulence traits shared with plant and human fungal pathogens. Proc Natl Acad Sci U S A 104: 18730-18735.

34. Martinez D, Challacombe J, Morgenstern I, Hibbett D, Schmoll M, et al. (2009) Genome, transcriptome, and secretome analysis of wood decay fungus Postia placenta supports unique mechanisms of lignocellulose conversion. Proc Natl Acad Sci U S A 106: 1954-1959.

35. Ohm RA, de Jong JF, Lugones LG, Aerts A, Kothe E, et al. (2010) Genome sequence of the model mushroom Schizophyllum commune. Nat Biotechnol 28: 957-963.

36. Kamper J, Kahmann R, Bolker M, Ma LJ, Brefort T, et al. (2006) Insights from the genome of the biotrophic fungal plant pathogen Ustilago maydis. Nature 444: 97-101.

37. Katinka MD, Duprat S, Cornillot E, Metenier G, Thomarat F, et al. (2001) Genome sequence and gene compaction of the eukaryote parasite Encephalitozoon cuniculi. Nature 414: 450-453.

38. Corradi N, Pombert JF, Farinelli L, Didier ES, Keeling PJ (2010) The complete sequence of the smallest known nuclear genome from the microsporidian Encephalitozoon intestinalis. Nat Commun 1: 77.

39. Akiyoshi DE, Morrison HG, Lei S, Feng X, Zhang Q, et al. (2009) Genomic survey of the non-cultivatable opportunistic human pathogen, Enterocytozoon bieneusi. PLoS Pathog 5: e1000261.

40. Ma LJ, Ibrahim AS, Skory C, Grabherr MG, Burger G, et al. (2009) Genomic analysis of the basal lineage fungus Rhizopus oryzae reveals a whole-genome duplication. PLoS Genet 5: e1000549.

41.Aury JM, Jaillon O, Duret L, Noel B, Jubin C, et al. (2006) Global trends of wholegenome

duplications revealed by the ciliate *Paramecium tetraurelia*. Nature 444: 171-

178.

42. Eisen JA, Coyne RS, Wu M, Wu D, Thiagarajan M, et al. (2006) Macronuclear genome

sequence of the ciliate *Tetrahymena thermophila*, a model eukaryote. PLoS Biol 4:

e286
