# Supplementary figures and images for: The Origin of GPCRs: Identification of Mammalian like Rhodopsin, Adhesion, Glutamate and Frizzled GPCRs in Fungi
Source: PLoS One. 2012 Jan 4;7(1):e29817. doi: 10.1371/journal.pone.0029817 (PMC3251606; doi:10.1371/journal.pone.0029817)

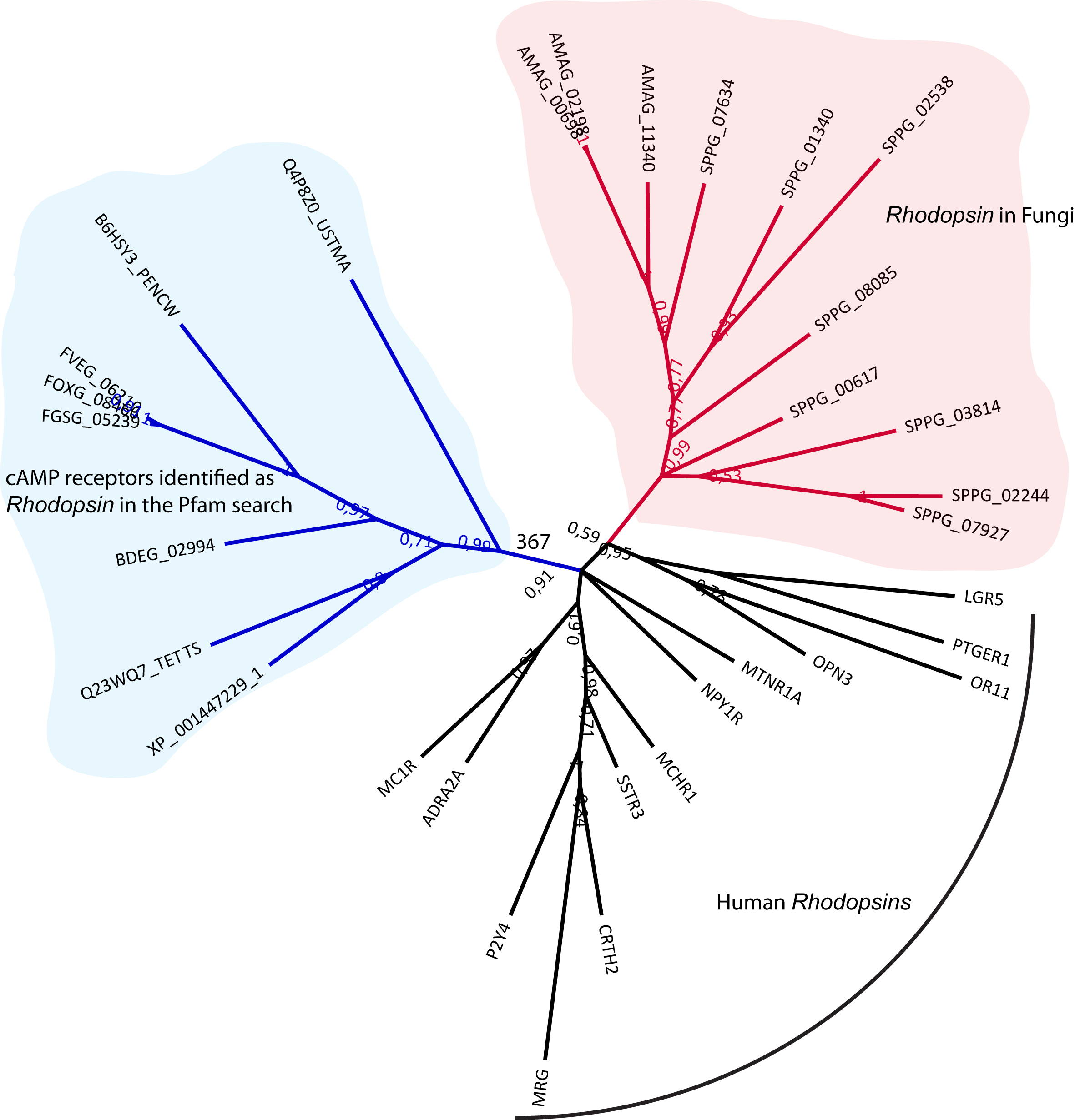

Supplement: Figure S1 — Phylogenetic relationship between the Rhodopsin family sequences in Fungi and human. (TIF) [file pone.0029817.s005.tif]

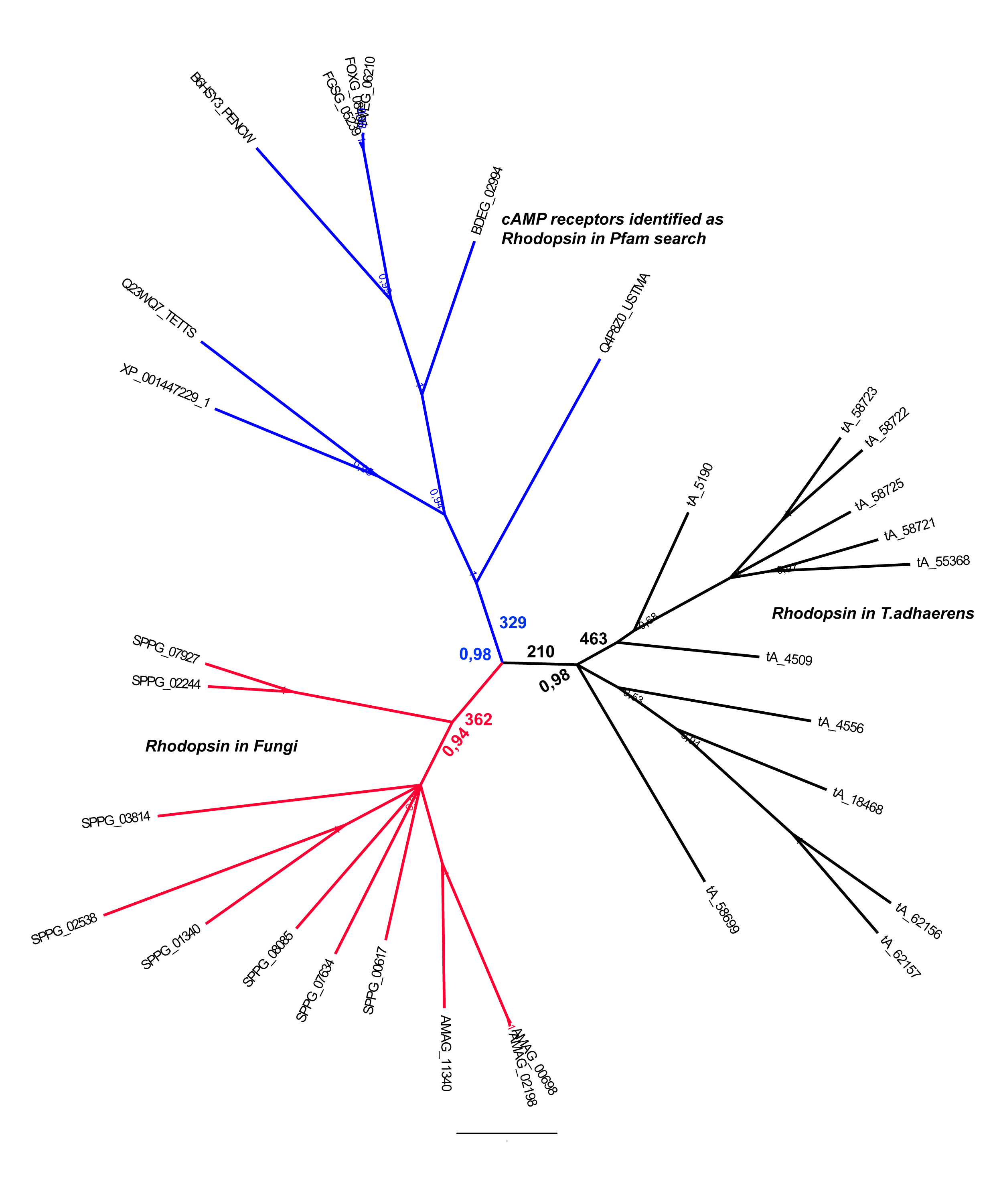

Supplement: Figure S2 — Phylogenetic relationship between the Rhodopsin family sequences in Fungi and T. adhaerens. (TIF) [file pone.0029817.s006.tif]

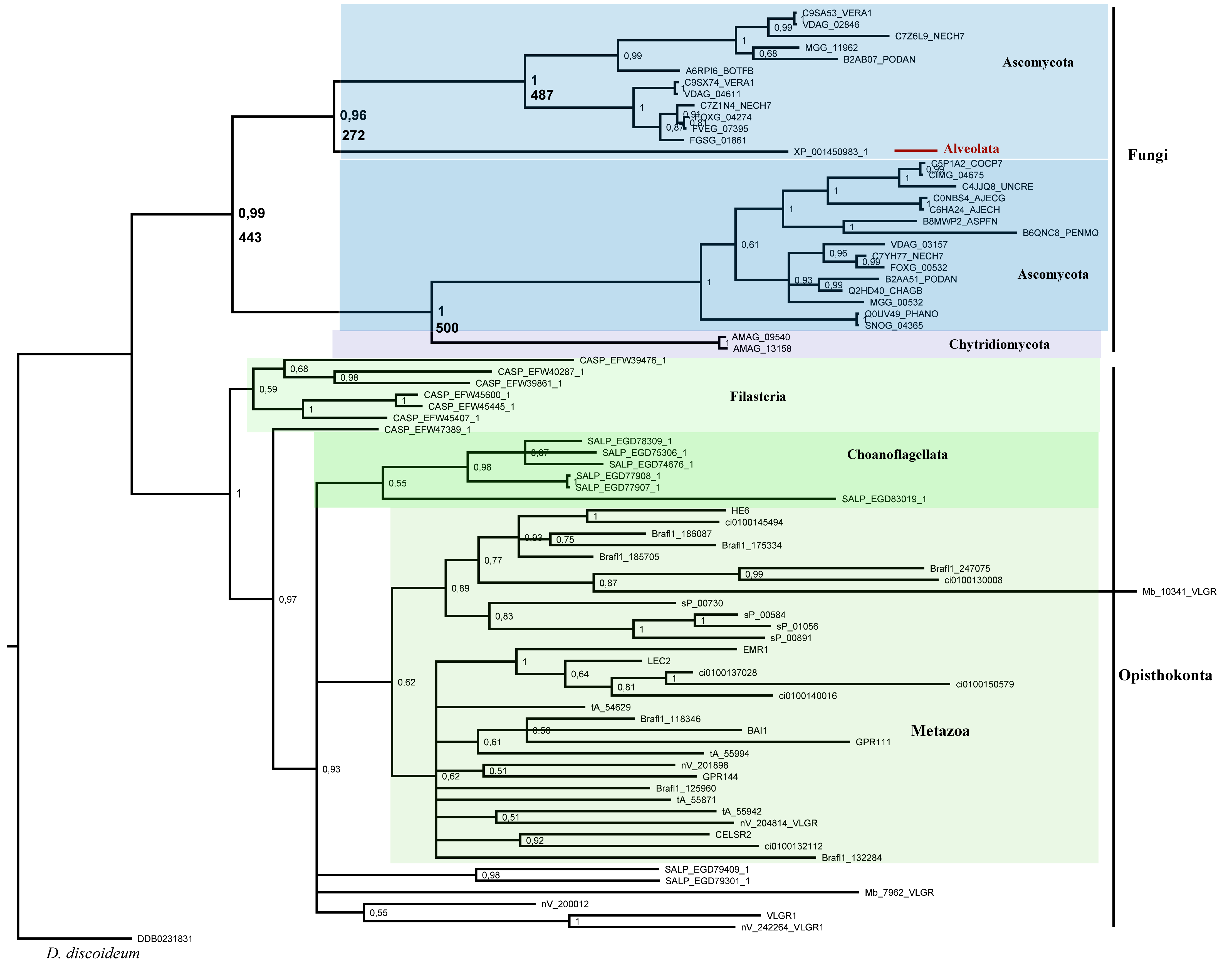

Supplement: Figure S3 — Phylogenetic relationship between the novel Adhesion family sequences in Fungi, Filasterea, Choanoflagellata and Alveolata with the representatives from Metazoa. The tree is rooted with D. discoideum. The node that is highlighted in red clustered the Adhesion receptor from Alveolata with the fungal members (PP>90%). They share about 50% identity within the 7TM regions (see Figure S8). (TIF) [file pone.0029817.s007.tif]

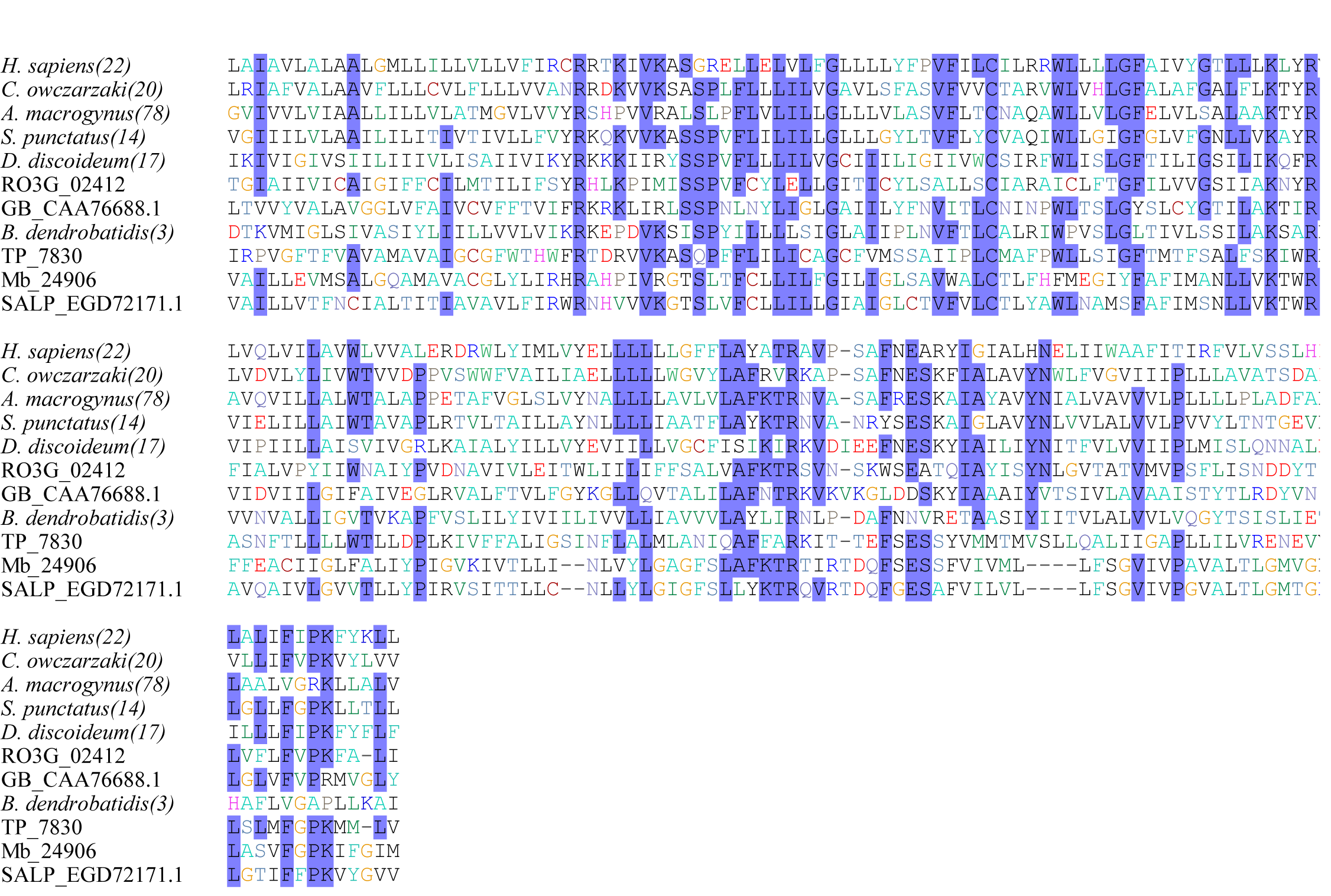

Supplement: Figure S4 — Alignment of the Glutamate family sequences in diverse eukaryotic lineages. The alignment shows the conserved regions within the 7TM region between the novel sequences in diverse eukaryotic lineages and human. The consensus sequence for each species was obtained from separate alignments. The number of sequences aligned to emit the consensus sequences for each species is given in the parenthesis. Regions which show >50% conservation are highlighted. (TIF) [file pone.0029817.s008.tif]

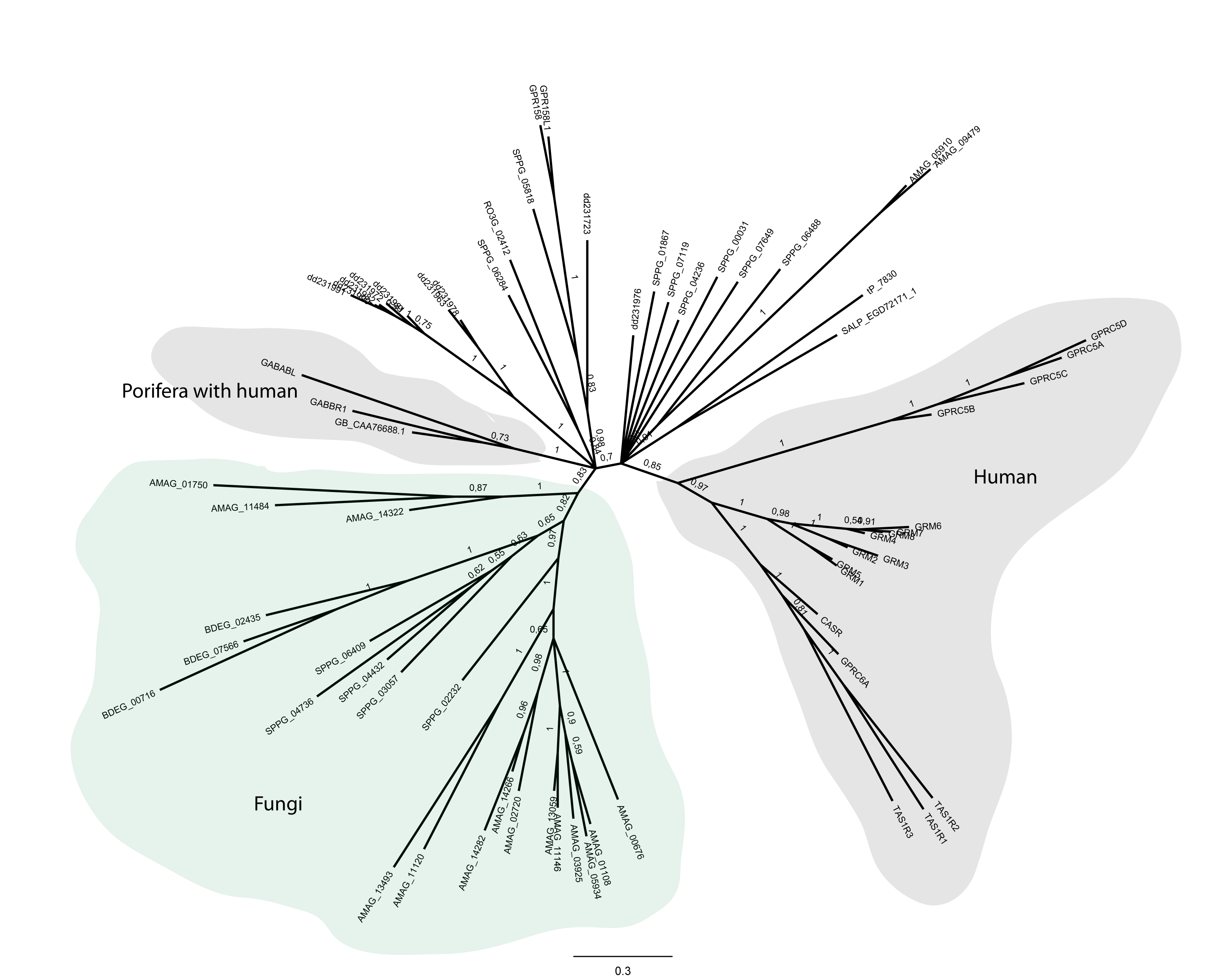

Supplement: Figure S5 — Phylogenetic relationship between the Glutamate family sequences in Fungi, Choanoflagellata, Porifera and Alveolata with human. (TIF) [file pone.0029817.s009.tif]

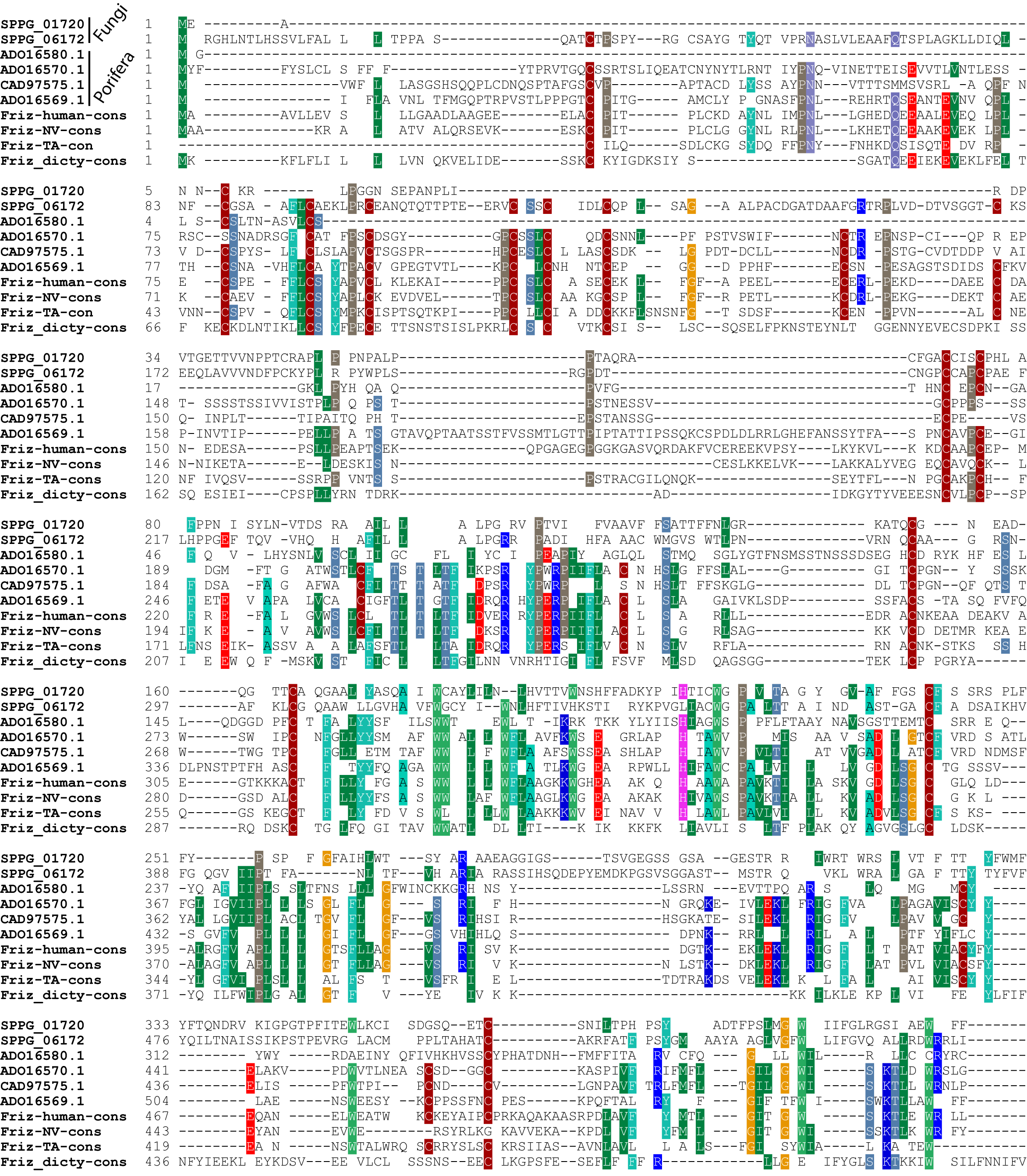

Supplement: Figure S6 — Alignment of the novel Frizzled receptor sequences in Fungi and Porifera with the representative consensus sequences of the Frizzled receptors from human, N. vectensis (NV), T. adhaerens (TA) and D. discoideum (dicty). Regions which show >50% conservation are highlighted. Multiple cysteine residues that are characteristic for the Frizzled family are mostly conserved in the novel sequences. (TIF) [file pone.0029817.s010.tif]

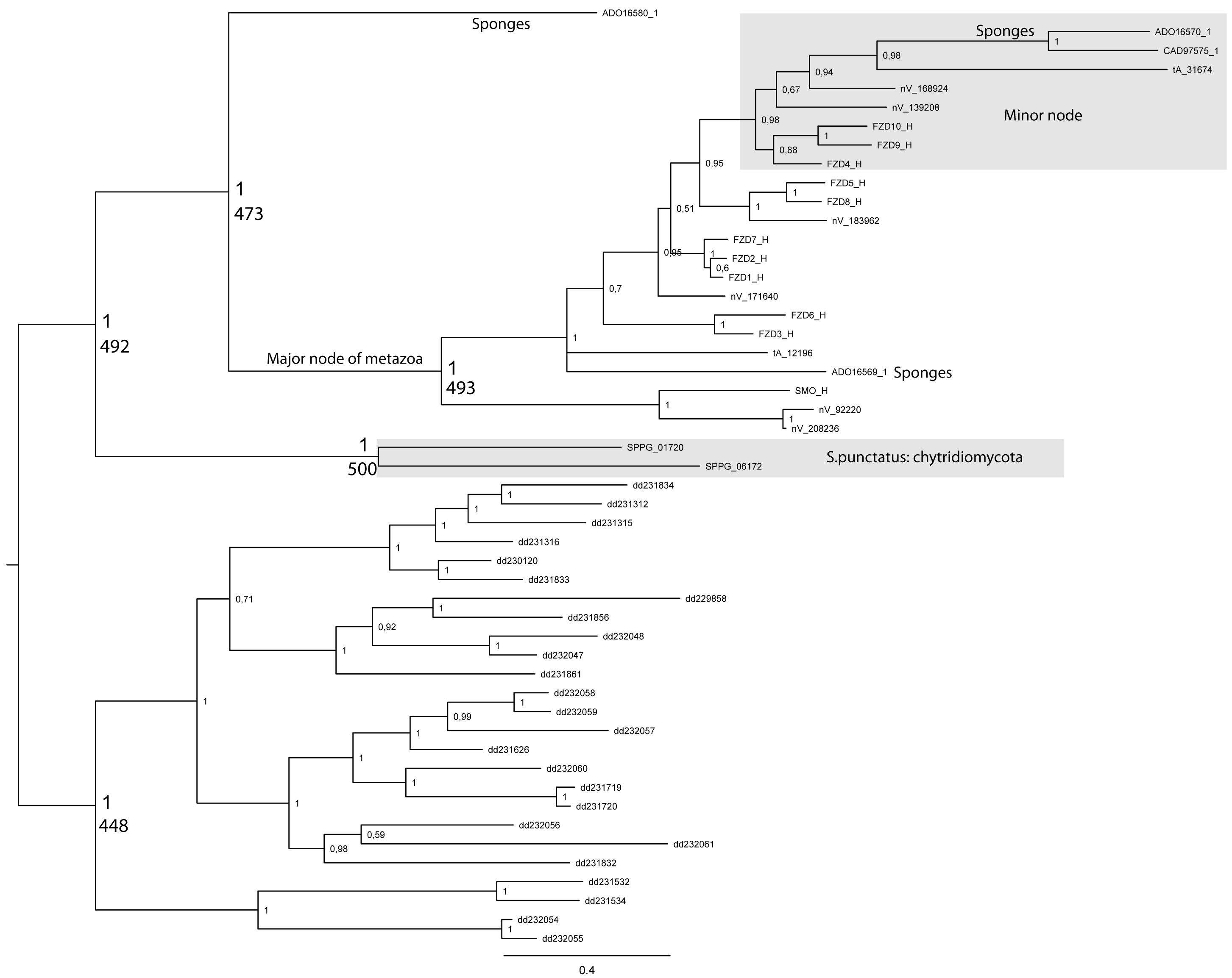

Supplement: Figure S7 — Phylogenetic relationship between the Frizzled family sequences in Fungi and sponges (Porifera) with the representatives from Metazoa. (TIF) [file pone.0029817.s011.tif]

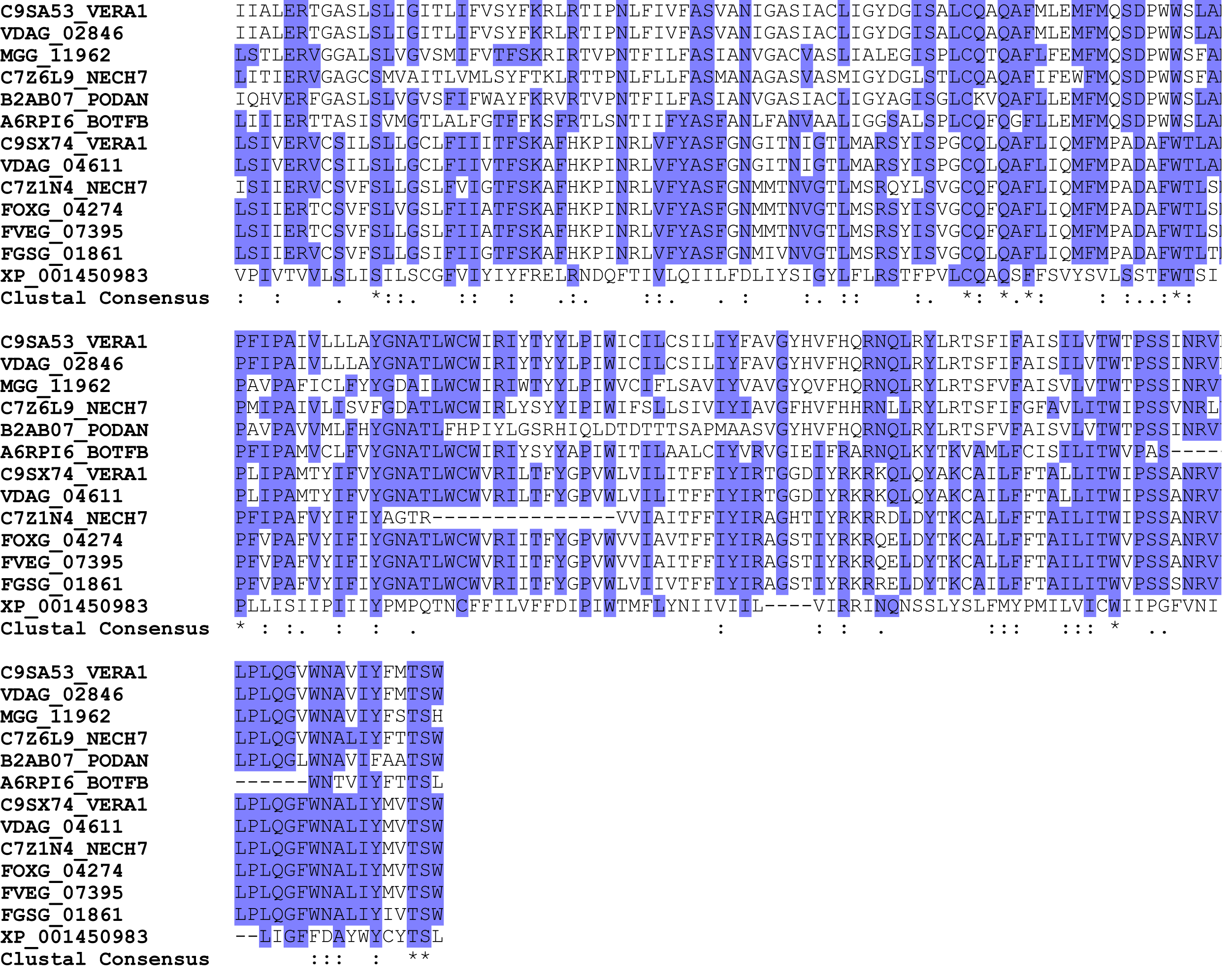

Supplement: Figure S8 — Alignment showing the conservation in the 7TM region between the Adhesion receptor sequences in Fungi and Alveolata. Regions which show more than 50% conservation are highlighted. (TIF) [file pone.0029817.s012.tif]
